# *De novo *assembly and characterization of a maternal and developmental transcriptome for the emerging model crustacean *Parhyale hawaiensis*

**DOI:** 10.1186/1471-2164-12-581

**Published:** 2011-11-25

**Authors:** Victor Zeng, Karina E Villanueva, Ben S Ewen-Campen, Frederike Alwes, William E Browne, Cassandra G Extavour

**Affiliations:** 1 Department of Organismic and Evolutionary Biology, Harvard University, 16 Divinity Avenue, Cambridge, MA 02138, USA; 2 Department of Biology, University of Miami, 234 Cox Science Center, 1301 Memorial Drive, Coral Gables, FL 33146, USA

## Abstract

**Background:**

Arthropods are the most diverse animal phylum, but their genomic resources are relatively few. While the genome of the branchiopod *Daphnia pulex *is now available, no other large-scale crustacean genomic resources are available for comparison. In particular, genomic resources are lacking for the most tractable laboratory model of crustacean development, the amphipod *Parhyale hawaiensis*. Insight into shared and divergent characters of crustacean genomes will facilitate interpretation of future developmental, biomedical, and ecological research using crustacean models.

**Results:**

To generate a transcriptome enriched for maternally provided and zygotically transcribed developmental genes, we created cDNA from ovaries and embryos of *P. hawaiensis*. Using 454 pyrosequencing, we sequenced over 1.1 billion bases of this cDNA, and assembled them *de novo *to create, to our knowledge, the second largest crustacean genomic resource to date. We found an unusually high proportion of C2H2 zinc finger-containing transcripts, as has also been reported for the genome of the pea aphid *Acyrthosiphon pisum*. Consistent with previous reports, we detected trans-spliced transcripts, but found that they did not noticeably impact transcriptome assembly. Our assembly products yielded 19,067 unique BLAST hits against **nr **(E-value cutoff e-10). These included over 400 predicted transcripts with significant similarity to *D. pulex *sequences but not to sequences of any other animal. Annotation of several hundred genes revealed *P. hawaiensis *homologues of genes involved in development, gametogenesis, and a majority of the members of six major conserved metazoan signaling pathways.

**Conclusions:**

The amphipod *P. hawaiensis *has higher transcript complexity than known insect transcriptomes, and trans-splicing does not appear to be a major contributor to this complexity. We discuss the importance of a reliable comparative genomic framework within which to consider findings from new crustacean models such as *D. pulex *and *P. hawaiensis*, as well as the need for development of further substantial crustacean genomic resources.

## Background

Crustaceans are one of the four major groups that make up the phylum Arthropoda, the most speciose and morphologically diverse animal group [1]. Despite the fact that arthropods as a whole make up the majority of animal species diversity and biomass, until recently the only arthropod represented in the list of NIH model organisms http://www.nih.gov/science/models/ was the fruit fly *Drosophila melanogaster*. The water flea *Daphnia pulex *was recently added to this list, and is the only crustacean to date with a publicly accessible sequenced genome [2]. As crustaceans are now widely recognized as sister group to the hexapods [3-7], the phylogenetic position of *D. pulex *suggests that it could serve as a useful outgroup to insects, providing meaningful comparisons with the many insights into developmental and disease biology provided by work on *D. melanogaster*. However, there are no genomic resources on a scale comparable to the *D. pulex *genome, and so it is still not known to what extent the characteristics of the water flea genome are specific to this animal's ecology or shared by other crustaceans [2]. Moreover, comparisons of biomedically relevant processes and mechanisms between *D. pulex *and other model organisms must be informed by robust phylogenetic hypotheses. At the moment, which specific subgroup of crustaceans is closest to the hexapods (including *D. melanogaster*) is still a matter of debate [8], but several phylogenetic hypotheses suggest that branchiopods may be more distant from insects than other crustacean groups [6,9-12].

Crustaceans have long been the subject of ecological and evolutionary study, as well as being lucrative commercial species for human consumption. Even for many of the most intensively studied of these [13], surprisingly few genomic projects have been reported. Most of the crustacean EST projects completed to date, notably for the farmed shrimp *Litopenaeus vannamei *(~163,000 ESTs) [14-16], the salmon ectoparasite copepod *Lepeophtheirus salmonis *(~129,000 ESTs) [17], the porcelain crab *Petrolisthes cinctipes *(~98,000 ESTs) [18], and the lobster *Homarus americanus *(~52,000 ESTs) [19-21], have all used Sanger sequencing of cDNA libraries. Next generation sequencing technologies are increasingly affordable, accessible and robust even for organisms lacking a sequenced genome [22], but have been reported to be applied to a crustacean *de novo *transcriptome only once, in the Antarctic krill *Euphausia superba *[23]. This organism is the subject of ecological and climate change research, but is not a viable laboratory model organism due to its specialized habitat.

While new understanding of developmental and molecular mechanisms in *D. pulex *are expected to follow from its genome sequence, it is important to note that crustaceans have been the subject of comparative embryology for over a century [24], and in recent decades, of evolutionary developmental biology ("evo-devo"). The morphological and molecular mechanistic variations of early embryogenesis, modifications of their body plans and appendage diversifications displayed by crustaceans have all been the subject of studies too numerous to describe here. However, until recently most comparative analyses of crustaceans have been limited to the study of gene expression or experimental embryology, as crustacean models where functional genetic testing is possible are still few in number. The limiting factor for comparative functional experiments is often obtaining specific coding sequences of sufficient length. A large-scale genomic resource for a model crustacean would therefore greatly facilitate development and deployment of transgenic tools.

The amphipod *Parhyale hawaiensis *has emerged as an important laboratory model crustacean species over the last several years [25]. *P. hawaiensis *was first described in the Hawaiian islands [24], but it occupies intertidal marine habitats worldwide. Laboratory husbandry is easy and affordable, and inbred lab cultures produce hundreds of embryos year-round, providing ample material for developmental studies. Fate map and cell lineage analyses of the early embryo show that all three germ layers and the germ line are determined by the eight cell stage [26], and clonal populations show predictable patterns at least up until gastrulation [27]. Despite this apparently "mosaic" embryonic development, significant regulative properties have also been described for the embryonic mesoderm and ectoderm [28]. Molecular techniques for the study of development, including stable transgenesis [29-31] and gene knockdown [32-34] are arguably better established for *P. hawaiensis *than for any other laboratory crustacean model.

However, the number of *P. hawaiensis *developmental genes available as GenBank accessions is less than 25. Progress in understanding the development of *P. hawaien*sis is thus is limited by the relative paucity of publicly available cloned coding and regulatory regions. Indeed, since the development of germ line transgenesis in *P. hawaiensis*, its use in developmental studies has been reported only three times [30,31,34].

Analysis of phenotypes of genomic transgene insertions [29] and case studies of intron sizes [35] are consistent with an extremely large genome size for this amphipod. Accordingly, the genome size of *P. hawaiensis *is estimated to be 2.98 Gb ([35], R. Gregory and C. Extavour, unpublished), very near that of *Homo sapiens *[36,37]. A genomic BAC library for *P. hawaiensis *has been created and partially sequenced [35] but is not yet available in GenBank. The genome of the closely related amphipod *Jassa marmorata*, with a much smaller genome of 690 Mbp (~4 times the size of the *D. melanogaster *genome) has been approved for whole genome, BAC end, and EST sequencing by the Joint Genome Institute [13], but this work is still ongoing, and laboratory culture of this amphipod is difficult. A large transcriptome dataset for *P. hawaiensis *would thus be a highly valuable resource for several scientific communities, and would in addition assist with the annotation of planned amphipod genomic projects. Recent construction of a *de novo *transcriptome for the milkweed bug *Oncopeltus fasciatus*, which also lacks a sequenced genome [22], suggested that 454 pyrosequencing would be a fruitful approach to obtaining a large scale *P. hawaiensis *transcriptome.

Here we present the *de novo *assembly of a maternal and embryonic transcriptome for *P. hawaiensis*, sequenced with 454 Titanium pyrosequencing. We describe particular features of the *P. hawaiensis *transcriptome that were revealed during assembly and annotation, including the presence of trans-splicing and enrichment for C2H2 Zn finger domain-containing transcripts. We annotate orthologues of genes involved in several major developmental patterning processes, gametogenesis in males and females, and members of major conserved animal signaling pathways. We observe a high proportion of apparently unique sequences in the transcriptome, and discuss these findings in the light of observations on the *D. pulex *genome and other existing crustacean genomic resources.

## Results and Discussion

### Collection and preparation of material for ovarian and embryonic cDNA libraries

**Figure 1:**
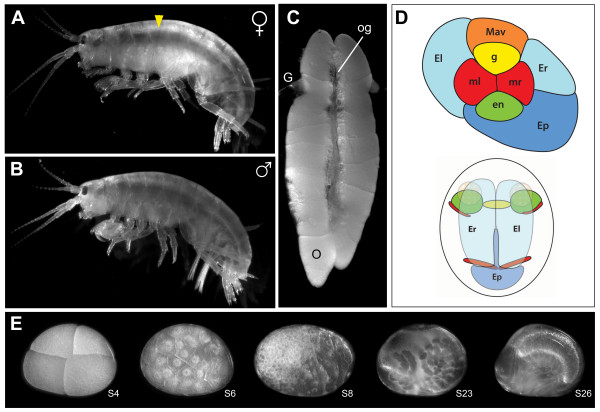
***Parhyale hawaiensis *and the tissues used to construct a *de novo *transcriptome**. (*A*) Adult female amphipod, *P. hawaiensis*. (*B*) Adult male. (*C*) Ovaries of adult female. Oocytes and oogonia are visible at various stages of growth. (*D*) Schematic drawings of the eight cell stage (top), at which all germ layers and the germ line are specified, and the germ band stage (bottom). Both of these signature stages are represented in this transcriptome. (*E*) A sample of the range of stages of *P. hawaiensis *embryogenesis represented in this transcriptome; stages as per [55]. Embryos from as early as S1 (one cell stage) and as late as S27 (just before hatching) were sampled; see Additional File 1 for details. G: gonoduct; O: late stage oocyte; og: younger oocytes and oogonia. Anterior is to the left in *A*, *B*, and *E*, and up in *C*.

Our goal was to create a transcriptome containing genes relevant to embryonic development, including both maternally provided and zygotically transcribed genes. All three germ layers and the germ line are determined by the eight cell stage [26], and we wished to capture transcripts from that early stage (Figure 1D top). We also wished to sample the intensively studied germ band stage (Figure 1D bottom), when major body axes have been patterned and trunk segmentation is ongoing [38], as well as later stages (S20-S27) when organogenesis is predominant. We therefore collected ovaries (Figure 1C) and embryos from all stages of embryogenesis (Figure 1E), extracted mRNA and prepared cDNA for 454 pyrosequencing. Because early stage embryos have many fewer cells than later stage embryos, we anticipated that transcripts present in early embryos might suffer low representation if our collection contained equal numbers of embryos of all stages. We therefore collected greater numbers of earlier stages than of later stages (Additional File 1).

### Sequencing and assembly of the *P. hawaiensis *transcriptome

**Figure 2:**
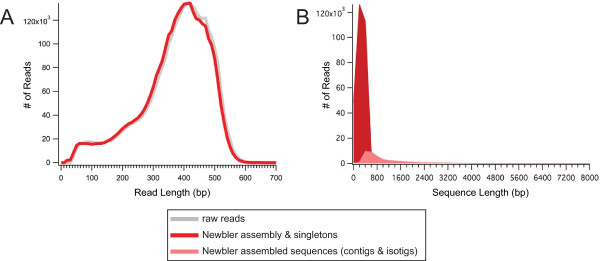
**Read lengths of raw, trimmed, and assembled reads**. (*A*) The raw reads (grey) ranged in length from < 40 bp to 1196 bp, with a mean read length of 400 bp. The distribution of read lengths of those reads chosen for assembly (red) was comparable to that of raw reads for the Newbler assembly (grey). (*B*) Read length distributions from all products of assembly of trimmed reads. The longest isotig per isogroup is shown. Removing singletons (unassembled reads) from these data shows that most assembly products under ~600 bp are singletons, i.e. that the vast majority of assembly products are transcript models over 600 bp (pink).

We sequenced a total of 3,172,925 reads (Table 1) with a median read length of 400 bp (Figure 2A; Additional File 2). For assembly of the *de novo *transcriptome we used Newbler v2.5 (Roche). Newbler's terminology for assembled reads distinguishes between contigs (groups of assembled reads with overlapping regions considered significant, i.e. putative exons), isotigs (continuous paths through a set of contigs, i.e. putative transcripts), and isogroups (groups of isotigs assembled from the same set of contigs, i.e. putative genes). For continuity, we use this terminology throughout this paper to refer to the products of the Newbler assembly.

**Table 1:** *P. hawaiensis *transcriptome assembly statistics

Raw reads (base pairs)	3,172,925 (1,179,544,291)
Assembled reads	3,157,373
Isotigs	35,301
Isotig N50	1,510
Singletons (% of assembled reads)	276,564 (8.8%)
# Unique BLAST hits	19,067
Isogroups ("genes")	25,735
Mean # isotigs per isogroup	1.4
Newbler Contigs ("exons")	89,664
Mean # contigs per isotig	2.1

The default parameters of Newbler v2.5 were used to screen for adapters and eliminate poor quality reads. The resulting 3,157,373 reads (99.5% of raw reads) were then assembled (Table 1). 2,349,266 (74.4%) of the screened reads were incorporated into assembled sequences (isotigs or contigs), with 276,564 (8.8%) singletons remaining. 531,543 reads (16.8% of reads subjected to assembly) were excluded because they were only partially assembled (431,372; 13.7%), from repeat regions (5,022; 0.2%), outliers (86,822; 2.7%), or too short (< 40 bp: 8,327; 0.3%). The assembly contained 89,664 contigs, which grouped into 35,301 isotigs. 18,615 (52.7%) of these isotigs were made up of only one contig, and the average number of contigs per isotigs was 2.1. The isotig N50 length was 1,510 bp, and the number of isogroups was 25,735 (18,565 (72.1%) of these comprised only one isotig, and the mean number of isotigs per isogroup was 1.4). The average coverage per contig was 7.1 reads/bp (Additional File 3). Averaging across all bases in the entire assembly, the average coverage per base pair was 25.4, meaning that every base pair in the transcriptome was sequenced 25.4 times on average. This coverage is high compared to typical numbers for *de novo *transcriptome assemblies, and should be helpful for distinguishing SNPs and indels from potential sequencing errors in raw reads [39]. Fasta files of all assembly products are freely available from the authors.

### BLAST mapping of non-redundant transcriptome sequences

Using an E-value cutoff of 1e-10, we first used BLASTX to map all non-redundant assembly sequences to **nr **(total number of sequences = 311,865 = 35,301 isotigs + 276,564 singletons), and obtained a total of 20,007 BLAST hits (Table 2). Of the 35,301 isotigs, 10,424 (29.5%) had at least one hit (including 9,715 contigs (10.8%), and of the 276,564 singletons, 9,583 (3.5%) had hits. The majority of these BLAST hits were unique: among the isotigs there were 10,203 (28.9%) unique hits, and among the singletons there were 8,864 (3.2%) unique hits (Table 2; see 'Sequence Annotation' within Materials and Methods for our determination of "unique BLAST hits"). In summary, among all non-redundant assembly sequences (i.e. isotigs + singletons), we obtained 19,067 unique BLAST hits (Table 2). These BLAST results may mean that the transcriptome contains as many as 19,067 unique gene transcripts. However, as for all *de novo *assemblies, several caveats must be considered. First, different regions of a single transcript may have different best top BLAST hits. Our assembly likely contains sequences that belong to the same transcript but are too far apart to be assembled together, and so would be considered "different genes." As a result, the number of unique BLAST hits may be an overestimate of unique gene number (see also discussion of total gene number estimation in section "*Comparison with other arthropod genomic resources*" below). Second, 24,877 isotigs (70.5% of all isotigs) and 266,981 singletons (96.5% of all singletons) did not yield BLAST hits that met our E-value cutoff of e-10. These values are comparable to or higher than those obtained in other *de novo *transcriptome analyses [40-45]. However, these unmatched sequences may represent transcript fragments whose similarities to known genes is too poor to meet our E-value cutoff, or are non-coding. It is therefore not formally possible from *de novo *assembly to know whether the 19,067 unique BLAST hit number over- or under-estimates the true number of genes contained in our transcriptome.

**Table 2:** *P. hawaiensis *transcriptome BLAST results

	# Sequences	**# BLAST hits against nr**^**1 **^**(%)**	**# Unique BLAST hits against nr**^**1 **^**(%)**
Isogroups	25,735	n/a^2^	n/a^2^
Isotigs	35,301	10,424 (29.5%)	10,203 (28.9%)
Contigs	89,664	9,715 (10.8%)	n/a^3^
Singletons	276,564	9,583 (3.5%)	8,864 (3.2%)
**Total Non-Redundant Assembly Sequences (= isotigs + singletons)**	**311,865**	**20,007 (6.4%)**	**19,067 (6.1%)**

1. nr = NCBI non-redundant database

2. Because isogroups are collections of isotigs that are hypothesized to originate from the same gene, they do not comprise a single sequence and so cannot be compared by BLAST to **nr**.

3. The custom script UniqueBlast.pl was applied only to non-redundant assembly sequences (i.e. isotigs and singletons).

### Transcriptome Gene Ontology (GO) term annotation

**Figure 3:**
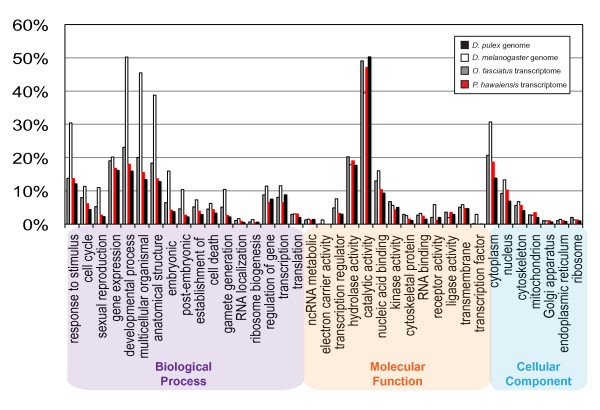
**GO term distribution of BLAST hits from the *P. hawaiensis *transcriptome**. For comparison, GO term distributions of transcript predictions from a crustacean (*Daphnia pulex*: water flea) and an insect (*Drosophila melanogaster*: fruit fly) with sequenced genomes are shown, as well as those from the largest available *de novo *pyrosequenced insect transcriptome to date (*Oncopeltus fasciatus*: milkweed bug [22]). Selected GO categories are shown within the top-level divisions of Biological Process, Molecular Function, and Cellular Component. Column heights reflect the percentage of annotated sequences that mapped to a given Biological Process GO term.

We used Blast2GO [46] to obtain the gene ontology terms associated with the top 50 BLAST hits for each non-redundant assembly sequence. Of the 19,067 sequences with unique BLAST hits as per our 1e-10 E-value cutoff, 9,451 (49.6%) of these had GO terms associated with them. To determine whether or not major categories of genes were missing or underrepresented in our transcriptome, we compared the proportions of sequences in selected GO term groups to the proportions in these categories observed in the predicted transcript complement of the only crustacean with a sequenced genome, *D. pulex *(see Materials and methods; Figure 3). We did not find significant differences in the proportion of genes in the examined GO term categories between the *P. hawaiensis *transcriptome and the water flea genome, suggesting that our transcriptome does not lack major functional categories of genes. Interestingly, not only were the proportions similar for the two crustaceans, they also closely matched the profiles observed for the sequenced genome of the insect *D. melanogaster*, and a *de novo *insect transcriptome from a milkweed bug [22] (Figure 3), suggesting that arthropod species as widely diverged as fruit flies and water fleas share similar proportional gene expression profiles in certain functional genetic categories.

### Unusual characteristics of the *P. hawaiensis *transcriptome

*P. hawaiensis *has been found to employ trans-splicing among its genetic regulatory mechanisms [47]. In this mechanism, sections of transcripts transcribed from independent genomic loci are spliced together post-transcriptionally to form a novel transcript. Such trans-spliced transcripts are recognizable because they contain a diagnostic splice-leader sequence. We wished to determine to what extent this additional transcript complexity affected our assembly and our ability to assign high-confidence BLAST annotations. We therefore processed the trimmed, pre-assembly reads to remove those containing the diagnostic splice-leader sequences (2,584; 0.1%), and performed a Newbler v2.5 assembly on the remaining reads. The results of this assembly were not noticeably different from the complete assembly, nor did the number of unique BLAST hits increase (Additional File 4). We therefore concluded that the presence of trans-spliced transcripts did not significantly affect our transcriptome assembly or annotation.

When we considered the species identity of the top BLAST hit for each isotig and singleton (see Methods), we found that a high proportion (50.5%) of our assembly sequences most closely matched sequences from other arthropods (Additional File 5). However, an unexpectedly high proportion (12.2%) was from the lancelet *Branchiostoma floridae*, which is phylogenetically very far removed from *P. hawaiensis*. When we examined those sequences with a lancelet sequence as top hit, we noticed that (a) for many of them, the top 50 BLAST hits were all *B. floridae*; and (b) the majority of them seemed to contain C2H2 zinc fingers with a specific linker sequence (TGEKP) between C2H2 domains.

**Figure 4:**
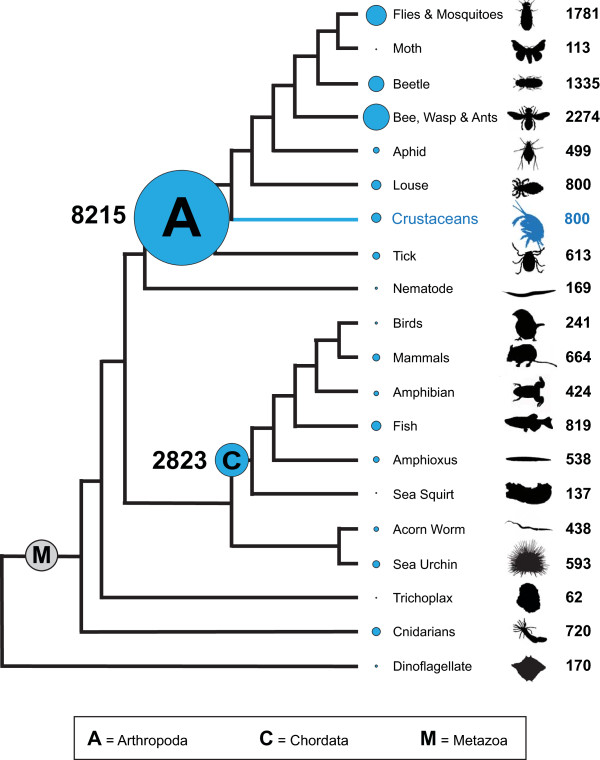
**Phylogenetic distribution of species of top unique BLAST hits for the *P. hawaiensis *transcriptome sequences**. First, raw reads that contained C2H2 zinc finger sequences were removed before assembly. Second, assembled sequences that contained C2H2 zinc finger sequences were removed from the output file. We then determined the unique BLAST hits for the remaining non-redundant assembly products (isotigs + singletons). 90% of the resulting unique BLAST hits were from species belonging to the clades shown. Blue circles are scaled according to the proportion of sequences with species belonging the clade indicated as their top BLAST hit. Over 50% of top BLAST hits are from arthropod species. Phylogenetic tree modified from [63,74,75]. Hits from the following most abundant species are represented: *D. mojavensis*, *D. willistoni*, *D. ananassae*, *D. grimshawi*, *D. pseudoobscura pseudoobscura*, *Aedes aegypti*, *Anopheles gambiae*, *Culex quinquefasciatus *(Flies & Mosquitoes), *Bombyx mori *(Moth), *Tribolium castaneum *(Beetle), *Harpegnathos saltator*, *Camponotus floridanus*, *Apis mellifera*, *Nasonia vitripennnis *(Bee, Wasp & Ants), *Acyrthosiphon pisum *(Aphid), *Pediculus humanus corporis *(Louse), *Ixodes scapularis *(Tick), *Penaeus monodon*, *Lepeophtheirus salmonis*, *Litopenaeus vannamei *(Crustaceans), *Caenorhabditis remanei *(Nematode), *Gallus gallus*, *Taeniopygia guttata *(Birds), *Rattus norvegicus*, *Mus musculus*, *Monodelphis domestica *(Mammals), *Xenopus laevis*, *X. tropicalis *(Amphibian), *Danio rerio*, *Tetraodon nigroviridis *(Fish), *Branchiostoma floridae *(Amphioxus), *Ciona intestinalis *(Sea Squirt), *Saccoglossus kowalevskii *(Acorn Worm), *Strongylocentrotus purpuratus *(Sea Urchin), *Trichoplax adherens *(Trichoplax), *Hydra magnipapillata*, *Nematostella vectensis *(Cnidarians), *Perkinsus marinus *(Dinoflagellate). See Additional File 5 for details.

The genome of at least one other arthropod, the pea aphid *Acyrthosiphon pisum*, has been found to be unusually rich in genes containing the same C2H2 zinc finger and diagnostic linker sequence [48]. We reasoned that if both the amphioxus [49] and amphipod genomes happened to contain a high proportion of such genes, this could be responsible for the apparent high similarity of several transcripts in these two species. To test this, we removed reads containing C2H2 zinc finger-encoding sequences, following a previously defined low-stringency definition of a C2H2 zinc finger domain [50]. We then assembled the remaining reads with Newbler v2.5, and again scanned for and removed contigs and isotigs containing the motif. The remaining assembly products were mapped against **nr **using BLASTX, and the species identities of their top hits were compared with those obtained from the complete assembly. We found that the new assembly retained a large proportion (54.3%) of arthropod hits, but that the number of *B. floridae *hits had dropped to 4.3%, and was now comparable to the proportion of hits obtained from other deuterostome phyla (Figure 4, Additional File 5).

### Comparison with other arthropod genomic resources

The BLAST hit rate of the *P. hawaiensis *isotigs was 29.5%. This is higher than those reported in other *de novo *transcriptome analyses [40-45], including that of an arctic crustacean [23], but much lower than the 43.4% obtained from a previously analyzed *de novo *insect maternal/embryonic transcriptome [22]. The ~70% of *P. hawaiensis *isotigs without a high confidence BLAST hit could be either *P. hawaiensis*-specific genes, or genes with relatively lower similarity to known genes. In addition, it is possible that many of our transcripts represent untranslated sequences rather than coding regions (for example, UTRs or noncoding RNAs), and therefore do not match **nr **protein sequences.

The relatively low proportion of hits may also reflect the fact that until recently (and including the version of **nr **we used for our analysis, which did not include the *D. pulex *genome at the time of analysis) very few crustacean sequences were included in **nr**. The *D. pulex *genome also contains a high proportion (~36%) of apparently *Daphnia*-specific genes, thought to be largely the result of amplification of selected gene families [2]. Noting that the E-value cutoff used for the *D. pulex *analyses (1e-5) was slightly more relaxed than that used in our initial analysis (1e-10), we repeated the BLASTX of our non-redundant *P. hawaiensis *transcriptome sequences against **nr **with an E-value cutoff of 1e-5. This yielded 26,494 unique BLAST hits (including 34.8% of isotigs and 5.7% of singletons). We therefore found that even adjusting the E-value cutoff to that used for characterization of the *D. pulex *genome, ~59% of the sequences in this *P. hawaiensis *transcriptome lack significant similarity to other characterized animal sequences. These observations suggest that crustaceans may have more species- or clade-specific genes than previously appreciated. Alternatively, these high numbers of apparently lineage-restricted genes may simply reflect the paucity of crustacean genomic resources currently in public databases.

Those *P. hawaiensis *transcriptome sequences which failed to obtain a significant BLAST hit when compared with **nr **might share more similarity with other crustaceans. To test this, we used BLAST to compare all *P. hawaiensis *transcriptome sequences that had failed our 1e-10 E-value cutoff against **nr**, with the predicted *D. pulex *transcriptome (see Methods) using BLASTX and a 1e-10 E-value cutoff. We then used BLAST to compare the obtained sequences with **nr **to determine their putative identities. We did not set an E-value limit for this second BLAST, in order to recover at least some minimal information about the identity of these genes (E-values for a subset of these, described below, are shown in Additional File 6). We found that 47.9% of these sequences came from arthropods, only 2.5% of which were crustaceans. The low crustacean representation in **nr **makes it difficult to obtain high confidence BLAST hits for this group. We therefore focused on determining what proportion of "**nr **orphan" *P. hawaiensis *sequences were highly similar to sequences from *D. pulex *by comparing the E-values for BLAST hits against both **nr **and against the *D. pulex *predicted transcriptome. We found that of the 423 *P. hawaiensis *sequences with higher similarity to *D. pulex *genes than to anything in **nr **(Additional File 6), 381 (90.1%) of these had E-values at least an order of magnitude higher for *D. pulex *compared to **nr**, and 30 of these (7.1% of total) had E-values greater than one for **nr **hits, but *D. pulex *hit E-values of 1e-11 or lower. Most of this "*Daphnia*-like" group matched arthropod sequences that were previously annotated as "hypothetical proteins," suggesting that non-insect crustacean sequence annotation could improve future annotation of the existing insect genomes.

The *D. pulex *genome has been found to contain a high number of genes (at least 30,907) [2]. Without a genome sequence for *P. hawaiensis*, we cannot accurately estimate gene number in order to perform a rigorous comparison with *D. pulex*. However, our transcriptome assembly identified 25,735 isogroups. Because isogroups are groups of isotigs assembled from the same set of contigs, isogroups may represent putative genes, with each isotig of the isogroup representing a transcript variant, for example a splice variant. We therefore speculate that *P. hawaiensis*, with a genome over one order of magnitude larger than that of *D. pulex *(C. Extavour & R. Gregory, unpublished), may also have a high gene number of at least 25,735 genes.

However, using isogroup number of this *de novo *transcriptome as a proxy for total gene number has two significant limitations. The first is the result of our chosen tissue sampling strategy: this transcriptome does not capture postembryonic gene expression. After hatching, expression of several genes with exclusively juvenile or adult roles is likely, including at minimum additional genes associated with molting, behavior, and gametogenesis. It is therefore possible that the number of isogroups in our assembly underestimates the true gene number in *P. hawaiensis*.

The second limitation is the unavoidable result of any *de novo *assembly, which is that if two sequences from the same transcript do not share significant overlap, they will appear as separate assembly products rather than as a single transcript (see also discussion of unique BLAST hit number in section "*BLAST mapping of non-redundant transcriptome sequences*" above). This could result in the number of isogroups being an overestimate of the true number of genes. A further complication results from Newbler v2.5's method of handling isogroups made of multiple isotigs. When we performed our assembly, we limited the number of isotigs in one isogroup to 10 ("-it" flag; see Methods). This has the advantage of avoiding isogroups composed of large numbers of isotigs, as we suspect that in *P. hawaiensis*, as shown for other animals, the vast majority of genes have fewer than ten splicing isoforms [51]. However, it can also result in problematic isogroup number calculation, because isogroups that exceed the number of isotigs per isogroup threshold are returned to the assembly file as contigs rather than isotigs, thus inflating the gene number estimate. In summary, in order to determine whether high numbers of species- or clade-specific genes is a general characteristic of crustaceans, and the true extent of species-specific genes for *P. hawaiensis*, more deep genomic resources will have to be developed for this amphipod and for more crustaceans of diverse classes.

**Figure 5:**
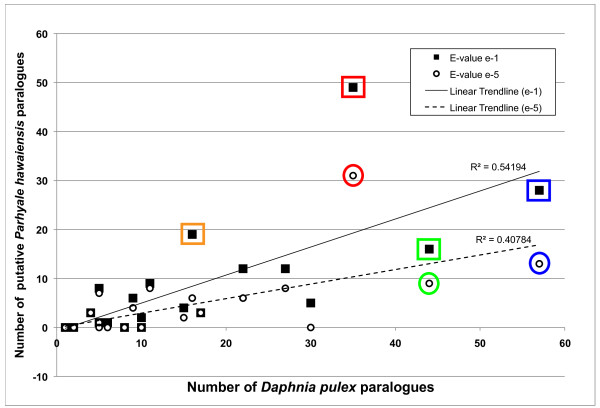
**Putative gene family expansion assessed with the *P. hawaiensis *transcriptome**. Gene families that were reported as expanded in the *D. pulex *genome [2] were examined for signatures of expansion in *P. hawaiensis *using the *de novo *transcriptome (see Methods). Gene families that appear particularly expanded in both crustaceans include chitinase (orange; KEGG EC number 3.2.1.14), adenosinetriphosphatase (red; KEGG EC number 3.6.1.3), 4-galactosyl-N-acetylglucosaminide 3-alpha-L-fucosyltransferase (green; KEGG EC number 2.4.1.152) and DNA-directed RNA polymerase (blue; KEGG EC number 2.7.7.6).

The high gene number of *D. pulex *is thought to be due to lineage-specific expansions of gene families [2]. These expansions may play adaptive roles in the water flea's ecology [2], or they may be a general feature of crustacean genomes that was previously unappreciated due to the paucity of crustacean genomic resources. It is therefore of interest to determine the extent of gene family expansion in *P. hawaiensis*. In order for our *de novo *transcriptome to provide a rigorous answer to this question, we would need to distinguish between transcripts of paralogues, and sequences originating from the same transcript that do not overlap enough to belong to the same isotig, or even obtain the same set of top BLAST hits. This distinction is not unambiguously possible, given the absence of a reference genome. However, we performed a preliminary analysis of putative gene expansion in *P. hawaiensis*, focusing on those gene families found to be expanded in the *D. pulex *genome. Our analysis conservatively included only those *P. hawaiensis *isotigs that had a top BLAST hit against a duplicated D. pulex gene. We also made sure that these isotigs had the same set of top BLAST hits but belonged to different isogroups, and were therefore likely to represent paralogues rather than splice variants (see Methods). We found that in general, highly expanded *D. pulex *gene families had more putative paralogues (isogroups) in *P. hawaiensis*, relative to less expanded gene families (Figure 5). It is therefore possible that gene family expansions are a common feature of certain enzymatic gene families in some crustaceans, although genome sequencing will ultimately be needed to provide definitive answers to this question.

### Assessment of depth and transcript coverage of the transcriptome

**Figure 6:**
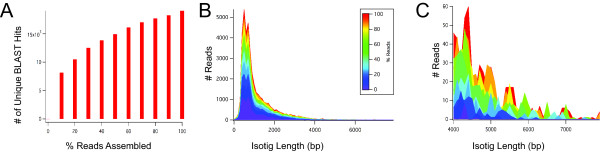
**Assessing complexity and depth of the *P. hawaiensis *transcriptome**. Randomly sampled subsets of increasing percentages of the total number of reads, in increments of 10%, were used to generate progressively larger sub-assemblies using Newbler v2.5. (*A*) The number of unique BLAST hits (performed against **nr**) continues to increase as more sequences from this sample are assembled. (*B*) Full range of isotig length distribution for each sub-assembly. The isotig length distribution remains similar across all sub-assemblies for the shorter (< 5000 bp) reads in the assembly. (*C*) High read length (> 4000 bp) range of assembled read length distribution for each sub-assembly Assembly of reads with length ≥ 5000 bp requires assembly of at least ≈60% of our reads, or ≈1.9 million reads. Legend shown in *B *applies to both *B *and *C*.

Although the 19,067 unique BLAST hits that we identified may represent unique genes, as discussed extensively above we cannot verify how many transcripts are encoded by the genome in the absence of an annotated genome sequence. However, we wished to estimate how deeply we had sequenced those transcripts that were present in our oogenesis/embryogenesis cDNA sample. To do this, we assembled progressively larger random subsets of the total reads. For each subassembly, we used BLAST to compare our non-redundant transcriptome sequences with **nr**, and assessed the number of unique BLAST hits to see to what extent adding sequence data from the same sample improved gene discovery. Even with the maximum number of over 3 million reads, we did not observe a plateau in the gene discovery rate (Figure 6A). This suggests that despite the considerable depth of our coverage, sequencing more reads from the same sample could yield even more new gene discovery. This is in contrast with a recently constructed *de novo *maternal and embryonic transcriptome for an insect, which was comprised of only 2 million reads and yet saturated gene discovery in the cDNA sample that was sequenced [22]. The increased complexity of the amphipod transcriptome may reflect its large genome size ([35], C. Extavour and R. Gregory, unpublished), high putative gene number (this study), or large predicted intron sizes [35].

Total isotig length increased steadily as progressively larger subsets of reads were assembled (Figure 6B). While small numbers of isotigs over 4,500 bp could be obtained with as few as ~300,000 reads, robust recovery of isotigs longer than 6,500 bp required assembly of at least 60% of our total reads, or ~1.9 million reads. This demonstrates that increased depth of sequencing, in addition to improving gene discovery, has the added benefit of increasing predicted transcript lengths, thereby facilitating their annotation and making them more immediately useful for downstream applications. In a related analysis, we searched the transcriptome for the presence of the small number of *P. hawaiensis *developmental genes available as GenBank accessions, and found that 52.4% (11/21) were present (Additional File 7). However, only for one of these genes (*Ph-prospero*) did our transcriptome add sequence data to the GenBank accession (Additional File 8). This may be a reflection of both the relatively rarity of these transcripts, and the fact that those genes identified to date have been the subject of intense developmental studies, and so sequences of considerable length have already been cloned to close to full length [30,34,52-57].

**Figure 7:**
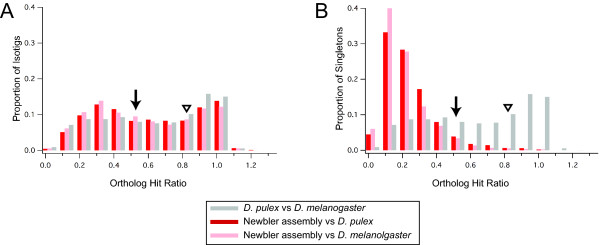
**Ortholog hit ratio analysis of assembled reads and singletons in *the P. hawaiensis *transcriptome**. As described in [39] an ortholog hit ratio of one suggests that a transcript has been assembled to its true full length. Ortholog hit ratios for two arthropod genomic datasets were obtained by using BLAST to compare the complete *Daphnia pulex *gene prediction set (downloaded from ftp://iubio.bio.indiana.edu/daphnia/genome/Daphnia_pulex/dpulex_jgi060905/fasta/dpulex-gnomon-transcript-jgi060905.fasta.gz.) with the predicted gene set of *Drosophila melanogaster *(r5.28 downloaded from ftp://ftp.flybase.net/genomes/Drosophila_melanogaster/) with an E-value cut-off of 1e-10. (*A*) Ortholog hit ratio analysis for isotigs. A majority appear to contain at least 50% of the full length transcript sequence (arrow) compared to *D. pulex *sequences (red), while over one third appear to represent at least 80% of the full length transcript sequence (arrowhead) compared to *D. pulex *sequences. Comparison with D. melanogaster transcripts (pink) yields comparable ortholog hit ratios. (*B*) Ortholog hit ratio analysis for singletons. Most singletons produced by both assemblers represent ≤ 20% of full-length transcripts. Arrow and arrowhead indicate 50% and 80% of full-length transcripts, represented by an average of 7.7% and 1.3% of singletons, respectively. In both panels, grey indicates comparison of *D. pulex *versus *D. melanogaster *transcripts based on predictions from genomic data.

The assembly yielded isotigs as long as 7,936 bp, with average length 1,128 bp (Figure 6B, C). However, we wished to determine what fraction of true full transcript length was likely to be contained by these isotigs. To do this, we used the methods of O'Neil and colleagues [39] in calculating the ortholog hit ratio for isotigs, contigs, and singletons. We found that 60.2% of isotigs represented over 50% of putative true full-length transcripts compared with predicted *D. pulex *transcripts, and 35.0% of isotigs were over 80% full length (Figure 7A). These ratios were not significantly higher than those obtained by comparing transcriptome sequences to *D. melanogaster *transcripts (58.1% and 33.2% respectively), suggesting that *P. hawaiensis *sequences have comparable similarity to those of the water flea and the fruit fly. Further, comparing transcripts from the fully sequenced genomes of *D. pulex *and *D. melanogaster *yields ortholog hit ratio values of 65.1% (above 0.5) and 41.7% (above 0.8) respectively, which are similar to the *P. hawaiensis*/*D. pulex *comparison values. These values are consistent with the increasing support for hypotheses of crustacean paraphyly, which predict large divergences between all of the lineages leading to these arthropod species [see for example 6]. The current state of understanding of crustacean-hexapod phylogenetic relationships (see Conclusions) therefore does not allow straightforward predictions of which pair of these three transcriptomes should be most similar to each other.

### Annotation of signaling pathway genes

**Figure 8:**
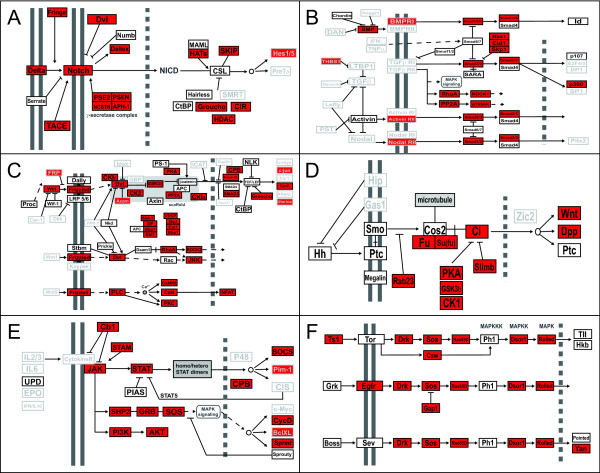
**Conserved metazoan signal transduction pathway components identified in the *P. hawaiensis *transcriptome**. These results were generated by using BLAST to compare sequences from known homologues against the full Newbler v2.5 assembly (see Additional File 9 and text for details), with genes identified marked in red. Genes outlined in grey with grey typeface indicate genes without *D. melanogaster *homologues. Pathway schematics modified from KEGG pathway model images http://www.genome.jp/kegg/kegg1.html. (A) Notch pathway. (B) TGFβ pathway. (C) Wnt pathway. (D) Hedgehog pathway. (E) Janus Kinase (JAK)-signal transducer and activator of transcription (STAT) pathway. (F) Mitogen-activated protein Kinase (MAPK) pathway.

Future functional genetic studies in *P. hawaiensis *will likely focus on elucidating the function of highly conserved metazoan genes in this amphipod. We therefore annotated the transcriptome for the presence of genes belonging to major conserved animal signaling pathways [58]. Using the KEGG pathways as a guideline [59], we searched for *P. hawaiensis *homologues of these genes using BLAST using an E-value cutoff of 1e-10. In most cases, the *D. melanogaster *homologue of a gene was used as a query, but for some searches, homologues from other organisms were used as queries (Additional File 9). For the Notch, TGFβ, Wnt, Hedgehog, JAK/STAT and MAPK pathways, considering pathway members known from all animals, we identified likely *P. hawaiensis *homologues of an average of 52.8% (103/195) of pathway genes (Figure 8). If we consider only those pathway members with known *D. melanogaster *homologues (n = 138), this proportion is an average of 74.6%. The proportion of genes found for each pathway ranged from 58.8% (MAPK pathway) to 93.8% (JAK/STAT pathway). Several genes of interest were found among the singletons. Although singletons are sometimes discarded before transcriptome annotation [see for example 23], our data suggest that even these unassembled reads can be a rich source of gene discovery. The transcriptome sequences for these genes ranged in length from 276 bp (*presenilin*, Notch pathway) to 4,882 bp (*CK2*, Wnt pathway), and the majority are at least 500 bp long, making them immediately useful for *in situ *hybridization, RNAi-mediated gene knockdown, and RACE [60] in the case that longer or flanking genomic sequences are required for specific applications. Interestingly, for several signaling pathway members without a *D. melanogaster *homologue, we found *P. hawaiensis *homologues (Figure 8), suggesting that in some respects, amphipod signaling pathways may bear greater resemblance to vertebrate pathways than fruit flies.

### Annotation of developmental and gametogenesis genes

Given the tractability of *P. hawaiensis *as a developmental model, we sought to make this transcriptome of maximal immediate use to the amphipod and broader developmental biology and biomedical research communities. We therefore undertook manual annotation of over 450 genes involved in embryonic patterning, developmental pathways (Additional File 10) and gametogenesis in both males and females (Additional File 11). We used lists of genes known to function in these processes in *Drosophila *as a starting point http://www.sdbonline.org/fly/aimain/1aahome.htm, and identified over 200 likely *P. hawaiensis *homologues of these genes. As we observed for our annotation of signaling pathway genes, the majority of matching transcriptome reads are of sufficient length to allow immediate use in gene expression and function studies.

Several of the genes contained multiple hits in our transcriptome, including a large number of singletons. For some genes, these multiple hits included consecutively numbered isotigs with very similar lengths. We examined a subset of these genes to determine whether or not these apparently highly similar isotigs were in fact different from each other. We found that such sequences usually differed from each other at a small number of nucleotide positions, either because of low sequencing quality in one of the component reads, or because of SNPs or small indels (Additional File 12). Although annotation of SNPs in this transcriptome is beyond the scope of this study, we anticipate that the SNPs present in this transcriptome could serve as a useful tool for population-level variation studies in the future.

## Conclusions

We have generated a maternal and embryonic transcriptome of the amphipod crustacean *Parhyale hawaiensis *using 454 Titanium pyrosequencing. To our knowledge, this represents the second largest crustacean genomic resource, behind the genome of the cladoceran *D. pulex*, and the largest *de novo *assembled pyrosequencing-based transcriptome to date. We confirmed the previously reported presence of trans-splicing in *P. hawaiensis *[47], but found that the presence of these trans-spliced transcripts did not greatly increase the complexity of the transcriptome or impede assembly. The *P. hawaiensis *transcriptome appears to be enriched for a particular class of C2H2 Zn finger-coding transcripts, which share high similarity with several *Branchiostoma floridae *transcripts, and is also reported to be an enriched sequence class in the genome of the pea aphid *Acyrthosiphon pisum *[48].

We annotated the *de novo *transcriptome for a large number of developmentally relevant genes, including major conserved metazoan signaling pathways. We found that even after assembly of over 3 million reads, gene discovery continued to increase, suggesting that the extremely large genome size of this amphipod may reflect high gene numbers, high transcript complexity, or both. These data should both facilitate future developmental and evolutionary studies using this emerging model crustacean species, and contribute to future work in crustacean comparative genomics.

The bulk of existing arthropod genomic resources are for insects, while the sister group to the insects, the crustaceans, remains relatively unexplored. The large genome size and low relative similarity to existing annotated arthropod genomes may be challenges to potential future efforts to sequence the *P. hawaiensis *genome. However, high throughput short read sequencing technologies such as Illumina or SOLiD [61], combined with the transcriptome described here, should make such projects tractable.

Finally, this dataset should assist phylogenomic approaches to resolution of controversies in crustacean phylogenetic relationships, including the relationship between crustaceans and hexapods. *D. pulex *belongs to the Branchiopoda, and while some phylogenetic analyses place this group closest to the hexapods [62-65], others suggest that the Malacostraca (the group including *P. hawaiensis *and most edible crustaceans such as shrimp, lobster and crab) are sister to the hexapods [4,9-11,66]. Yet others suggest that Branchiopoda may be the most derived group within a monophyletic clade containing all crustaceans except for Malacostraca, Remipedia and Cephalocarida [67]. The largest phylogenomic assessment of this problem to date places both Branchiopoda and Malacostraca in a monophyletic clade that is sister to a (Hexapoda + Remipedia + Cephalocarida) clade [6]. Moreover, *Daphnia *species do not branch basally within the Branchiopoda, and indeed are placed in the most derived branchiopod clade by most phylogenetic analyses [6,68,69]. These competing hypotheses emphasize the importance of a reliable comparative genomic framework within which to consider findings from new crustacean models. In order to place future comparative, ecological, environmental and biomedical studies using crustacean models in an appropriate phylogenetic context, additional crustacean genomic resources will be necessary.

## Methods

### Animal culture

The *P. hawaiensis *(Figure 1A, B) specimens sequenced in this study were from an inbred, non-backcrossed, non-isogenic laboratory culture originally obtained from Ernst Wimmer in 2002; the Wimmer culture in turn was obtained from a laboratory culture from Nipam H. Patel that was established with animals from the John G. Shedd Aquarium (Chicago, IL) as previously described [55]. The animals were maintained in the laboratory in artificial seawater (Instant Ocean, specific gravity 1.018-1.022), and fed a mixture of raw carrots, TetraAlgae Vegetable Enhanced Crisps, TetraMin Tropical Flakes, and Hikari Wheat Germ Pellets. All cultures were maintained under a 12:12 light/dark cycle at 28°C.

### cDNA Synthesis

471 mixed-stage embryos (Additional File 1; total weight 52.7 mg) representing the entirety of embryogenesis (Figure 1D) were shock frozen in liquid nitrogen and stored at -80°C. 30 ovaries (comprising eight small ovaries from young females and 22 late ovaries containing mature oocytes from older females; Figure 1C) were dissected from females in Trizol, flash frozen in Trizol, and stored at -80°C. Total RNA was isolated separately from ovaries (Figure 1C) and from mixed stages of embryogenesis (Figure 1E), and a pool was created of 1.5 μg of total RNA from each sample for use as a template for first strand cDNA synthesis (3 μg total). cDNA was synthesized following a protocol developed specifically for 454 Titanium sequencing of cDNA [22], with the exception that none of the cDNA was normalized in the present study. This protocol is based on the SMART cDNA library construction kit (Clontech, CA, USA), and includes a modified poly(T) primer to enrich for mRNA, and a DNAse treatment step to remove possible genomic DNA contamination. Following first strand cDNA synthesis, primary amplification of the cDNA required thirteen PCR cycles to maximize yield while avoiding overcycling (monitored in real-time via qPCR [22]). Secondary amplification began to plateau after nine cycles. To obtain sufficient double-stranded cDNA for pyrosequencing sequencing (~5 μg) without overcycling, 26 reactions of 100 μg each were run in parallel and subsequently co-purified into 90 μl of elution buffer using QIAquick PCR purification columns (Qiagen Inc).

### 454 Titanium Pyrosequencing

The samples were nebulized, adaptor-ligated, and pyrosequenced using the GS-FLX Titanium platform by the Institute for Genome Science and Policy DNA Sequencing Facility (Duke University). All of the raw reads generated in this study have been submitted to the NCBI Short Read Archive (Study Accession Number: SRA021010).

### Sequence Assembly

Raw reads were assembled using the cDNA assembly algorithm (the "-cdna" flag) of Newbler v2.5. An adaptor-trimming step was included in the assembly (the "-vt" flag). Screening adaptors used are available at http://extavourlab.com/protocols/ExtavourLab_454_Adapters.fasta). A vector-screening step (the "-vs" flag) was performed using a FASTA version of the Univec database ftp://ftp.ncbi.nih.gov/pub/UniVec/. Due to the long average length of the raw 454 reads, the minimum overlapping length (the "-ml" flag) was set to the default value of 40 base pairs. To accommodate the possible existence of SNPs and pyrosequencing errors, the minimum identity between sequences (the "-mi" flag) was set to require 95% identity between aligned sequences. Trimmed singleton reads were specifically produced by Newbler (the "-trim" flag). A maximum isogroup size of 10 isotigs was specified (the "-it" flag) to prevent Newbler from constructing an isogroup using an overly large quantity of short contigs. The resulting assembled reads and unassembled singletons were used for all subsequent analyses.

### Sequence Annotation

Sequences were first mapped against the **nr **(all non-redundant GenBank CDS translations+PDB+SwissProt+PIR+PRF) peptide sequence database [70, downloaded from ftp://ftp.ncbi.nih.gov/blast/db/on April 27, 2010] using BLASTX. Unless otherwise specified, all BLAST searches were conducted using BLAST v2.2.24+ [71] with an E-value cutoff of 1e-10.

To annotate Gene Ontology (GO) terms [72] and their parents associated with the top 50 BLAST hits for each sequence, we used Blast2GO v1.2.7 [46].

Due to the sequence depth of this project, the transcripts were assembled with the raw 454 reads were not all full-length. If sequences represent different regions of the same transcript that are not assembled together due to insufficient overlap, they run the risk of being counted twice in our BLAST annotation. To address this problem, a custom Perl script was created in order to estimate the number of unique transcripts present in the *P. hawaiensis *transcriptome (called "UniqueBlast.pl" available at http://www.extavourlab.com/protocols/bio_tools/Perl_Transcriptome_Analysis_Scripts.zip). UniqueBlast.pl utilizes the results of BLASTX against **nr **for all assembly products with an E-value cutoff of 1e-10, to predict whether multiple assembly products are fragments of the same transcript. UniqueBlast.pl accomplishes this by comparing the HSP region of assembly products with the same top BLAST hit. Assembly products that have the same top BLAST hit but non-overlapping HSP regions are considered as fragments of the same transcript. Assembly products with the same top BLAST hits and overlapping HSP regions are considered as putative isoforms or paralogues of each other. Overlapping is defined as greater than 14 amino acids shared within the HSP, because the assembly parameters require a minimum of 40 matching nucleotides in order for two raw reads to be assembled together. UniqueBlast.pl then generates a list of assembly sequences (isotigs or contigs) showing the longest fragment of each transcript, as well as predicted isoforms and paralogues. This list of unique transcripts was then used for all subsequent analysis including GO analysis, ortholog hit ratio calculations, and phylogenetic distribution analysis of top BLAST hit organisms.

For analysis of the phylogenetic distribution of species of top unique matches to non-redundant assembly sequences, we used BLASTX to compare our non-redundant assembly products (isotigs + singletons, see Table 2) with **nr **using an E-value cutoff of 1e-10. We discarded redundant hits as described using "UniqueBlast.pl." For each gene in this list of unique BLAST hits, we recorded the species identity of the top BLAST hit sequence. We then tallied the total numbers for clades of interest, shown in Figure 4 and Additional File 5. When assessing the effect of *Branchiostoma floridae *C2H2 Zn finger sequences on the annotation of the assemblies, we assessed top BLAST hits obtained with both the NCBI reference Sequence collection (RefSeq) and **nr**, and obtained comparable results for both searches.

For comparison of sequences belonging to different GO terms between species, we used the *Oncopeltus fasciatus *data from a previously generated transcriptome [22] and a precomputed GO annotation of the *D. melanogaster *genome [73]. To obtain GO category data for *Daphnia pulex*, we used the transcripts predicted from the *D. pulex *genome (downloaded on 3 December 2010 from ftp://iubio.bio.indiana.edu/daphnia/genome/Daphnia_pulex/dpulex_jgi060905/fasta/dpulex-gnomon-transcript-jgi060905.fasta.gz).

To search for developmental genes of interest, we used TBLASTN with protein queries being the full length *Drosophila melanogaster *homologue of the gene of interest. For genes that yielded no hits when the *D. melanogaster *homologue was used as a query, homologues from other animals were used as queries. In cases where proteins possessed domains that were also shared by other genes from a different gene family (e.g. *abstrakt*: zinc finger domain), the TBLASTN search was performed by masking the relevant domain in the query sequence. Finally, in cases of genes with small, diagnostic conserved domains within an otherwise poorly conserved sequence, the diagnostic domains were used as the query (e.g. *groucho*). An E-value cutoff of e-10 was used for all BLAST searches, except for those searches with masked domains or specific protein domains, in which case the E-value cutoff was e-5. For all developmental genes found, species identity and domain details of the query used are indicated in the legends to Additional Files 9, 10 and 11.

### Removal of reads from trans-spliced sequences

To determine how the presence of trans-spliced sequences affected our assembly with Newbler, we used the *P. hawaiensis *splice leader sequences [as per Figure 1 of 47] as an "adapter" sequence (the "-vs" flag) in the trimming step performed by Newbler prior to assembly. This resulted in removal or trimming of all raw reads containing the splice leader sequence. The remaining reads were assembled with Newbler v2.5 and compared using BLAST against **nr **as described for the complete assembly.

### Removal of C2H2 Zinc finger-containing sequences

Because we suspected that specific characteristics of *P. hawaiensis *C2H2 Zn-finger containing proteins might be responsible for a high incidence of BLAST hits to sequences of the lancelet *Branchiostoma floridae*, we performed a new assembly after removing a subset of reads in the following way: we scanned all reads for the presence of C2H2 Zn finger-encoding sequences using the least stringent C2H2 motif defined in [50], which is X_2_-C-X_1,2,4,5_-C-X_12_-H-X_3-6_-(H,C). We used this least stringent criterion rather than the most stringent criterion defined by Böhm and colleagues[50], in order to capture the largest number of reads containing these motifs. We reasoned that even "C2H2-like" domains might result in a Zn finger BLAST match, thus skewing the proportions of *B. floridae *hits. We used using a custom script ("C2H2.pl" available at http://www.extavourlab.com/protocols/bio_tools/Perl_Transcriptome_Analysis_Scripts.zip) and removed reads with matches. The remaining reads were assembled with Newbler v2.5, and the resulting assembly was scanned again for the presence of C2H2 Zn finger-encoding sequences; assembled reads with hits were discarded. The remaining sequences were compared with **nr **using BLAST.

### Estimating sequencing depth and transcript completion

To determine to what extent we had saturated gene discovery in the libraries we sequenced, we performed independent assemblies of ten progressively larger, randomly sampled subsets of the reads. The total number of genes in each sub-assembly was then identified via BLASTX against **nr**. If multiple isotigs or contigs hit non-overlapping portions of the same top BLAST hit, only one of these sequences was counted. To calculate the ortholog hit ratio [39], we first used the script for generating a list of unique BLAST results described above ("UniqueBlast.pl"). We then used a custom ortholog hit ratio script ("OrthologHitRatio.pl" available at http://www.extavourlab.com/protocols/bio_tools/Perl_Transcriptome_Analysis_Scripts.zip) to calculate the values used to create the graphs in Figure 6.

### Estimating extent of gene family expansion

We first identified the *D. pulex *transcripts belonging to the duplicated gene families described by Colbourne and colleagues et al (Figure S31 in [2]) based on the KEGG enzyme code. We recorded the NCBI Gnomon transcript prediction ID of each *D. pulex *transcript that was listed with the chosen KEGG enzyme codes. We then mapped all of the *P. hawaiensis *isotigs against the *D. pulex *Gnomon-predicted transcriptome using TBLASTN. All *P. hawaiensis *isotigs with a top BLAST hit matching any recorded *D. pulex *NCBI Gnomon transcript prediction ID were identified. If the *P. hawaiensis *isotigs identified in this way belonged to the same isogroup, only a single isotig from that isogroup was counted. Using this method, we counted the putative number of *P. hawaiensis *paralogues from a chosen gene family.

[The sequence data from this study have been submitted to GenBank under study accession number SRA021010. Custom scripts generated are available at http://www.extavourlab.com/protocols/bio_tools/Python%20Transcriptome%20Analysis%20Tools.tar.gz and http://www.extavourlab.com/protocols/bio_tools/Perl_Transcriptome_Analysis_Scripts.zip. Assembly results are available at http://www.extavourlab.com/resources/index.html and at http://www.bio.miami.edu/wbrowne/BrowneLab2/Community_Resources.html.]

## Authors' contributions

VZ performed experiments, helped design data analysis and analyzed data. KEV performed experiments and analyzed data. BEC helped design research, performed experiments, collected and analyzed data. FA performed ovary dissections and embryo collections. WEB helped design the research, performed experiments, analyzed data and obtained funding for the research. CGE proposed the idea for the research, helped design the research and analyze the data, wrote the manuscript with input from all authors, and obtained funding for the research. All authors read and approved the final manuscript.

## Supplementary Material

Additional file 1 **Embryonic stages pooled for creation of the *P. hawaiensis *transcriptome**. Staging as per [55]. Click here for file

Additional file 2 **Comparison of read lengths from Newbler v2.5 *de novo *assembly of the *P. hawaiensis *transcriptome**. (*A*) Distribution of read lengths after assembly with Newbler v2.5 (red). (*B*) Distribution of read lengths of the shortest assembled reads and raw reads. The assembly yielded assembled reads of over ~4000 bp. Click here for file

Additional file 3 **Distribution of average coverage (reads/bp) within contigs produced by Newbler v2.5 *de novo *assembly of the *P. hawaiensis *transcriptome**. The coverage within contigs is calculated by dividing the total number of base pairs contained in the reads used to construct a contig by the length of that contig. Click here for file

Additional file 4 **Analysis of the effect of trans-splicing transcripts on *de novo *transcriptome assembly**. Assembly of all trimmed sequences compared to assembly of sequences lacking the trans-splicing leader sequences [47]. Number of BLAST hits reflects a search against the **nr **database with an E-value cut-off value of 1e-10. Click here for file

Additional file 5 **Phylogenetic distribution of species of top unique BLAST hit for Newbler v2.5 assembly of the *P. hawaiensis *transcriptome**. Of the unique BLAST hits to all non-redundant assembly products (isotigs + singletons), 90% were from species belonging to the clades shown. Over 50% of these top BLAST hits are from arthropod species. The large number (12.2%) of top BLAST hits in the complete assembly to sequences from *Branchiostoma floridae *is due to the high similarity of C2H2 zinc finger domain-containing sequences with a particular linker sequence (TGEKP) that is also highly represented in the genome of the aphid *Acyrthosiphon pisum *[48]. Red: values after removal of reads and sequences containing this domain. Phylogenetic tree modified from [63,74,75]. Hits from the following most abundant species are represented: *D. mojavensis*, *D. willistoni*, *D. ananassae*, *D. grimshawi*, *D. pseudoobscura pseudoobscura*, *Aedes aegypti*, *Anopheles gambiae*, *Culex quinquefasciatus *(Flies & Mosquitoes), *Bombyx mori *(Moth), *Tribolium castaneum *(Beetle), *Harpegnathos saltator*, *Camponotus floridanus*, *Apis mellifera*, *Nasonia vitripennnis *(Bee, Wasp & Ants), *Acyrthosiphon pisum *(Aphid), *Pediculus humanus corporis *(Louse), *Ixodes scapularis *(Tick), *Penaeus monodon*, *Lepeophtheirus salmonis*, *Litopenaeus vannamei *(Crustaceans), *Caenorhabditis remanei *(Nematode), *Gallus gallus*, *Taeniopygia guttata *(Birds), *Rattus norvegicus*, *Mus musculus*, *Monodelphis domestica *(Mammals), *Xenopus laevis*, *X. tropicalis *(Amphibian), *Danio rerio*, *Tetraodon nigroviridis *(Fish), *Branchiostoma floridae *(Amphioxus), *Ciona intestinalis *(Sea Squirt), *Saccoglossus kowalevskii *(Acorn Worm), *Strongylocentrotus purpuratus *(Sea Urchin), *Trichoplax adherens *(Trichoplax), *Hydra magnipapillata*, *Nematostella vectensis *(Cnidarians), *Perkinsus marinus *(Dinoflagellate). Click here for file

Additional file 6 **Sequences with strong similarity to *Daphnia pulex *gene sequences identified in the *de novo P. hawaiensis *transcriptome**. Because the *D. pulex *genome and **nr **are databases of inevitably different sizes, E-values shown here are for information only and are not strictly comparable. See text for additional details. Click here for file

Additional file 7 **Presence of existing *P. hawaiensis *GenBank accessions in the *de novo *transcriptome**. Sequences of *P. hawaiensis *developmental genes from GenBank were used as a query to BLAST the *de novo *transcriptome. Most genes with hits had several matches in the transcriptome, among both assembled reads and singletons. Click here for file

Additional file 8 **The *P. hawaiensis *transcriptome adds sequence data to GenBank accession number HM191476, the *P. hawaiensis prospero *homologue**. Extended contig for *Ph-prospero*, comprising the complete mRNA GenBank accession (top, light grey), one isotigs and one contig from the Newbler assembly of the transcriptome (dark grey). The isotig provides an additional 445 bp of 3' UTR sequence and 116 bp of 5' UTR sequence (black) to the GenBank sequence. Comparison with the GenBank sequence shows that isotig24415 and singleton GAP9EXG06HFGHB belong to the same contig. Click here for file

Additional file 9 **Selected signaling pathway genes identified in the *P. hawaiensis *transcriptome**. Hit ID indicates if gene hits were found assembled reads (A) or singletons (S). Sequence length (range) indicates the shortest and longest A or S hit sequences for each gene. These results are shown graphically in Figure 7. Groups of hits of a given colour indicate transcriptome sequences that mapped to the same overlapping region of the BLAST target; hits of different colours indicate transcriptome sequences that map to different, non-overlapping regions of the BLAST target. Query organisms: *Dm *= *D. melanogaster*; *Dr *= *Danio rerio*; *Xt *= *Xenopus tropicalis*. Query sequence details: 1. Kinase domain was masked. 2. FERM domain used as query. 3. Amino acids 500-833 (Dl/Ser domain) used as query. 4. Amino acids 1-250 (groucho/TLE domain) used as query. 5. Kinase domain masked; amino acids 420-1390 used as query. 6. Kinase domain masked; amino acids 175-372 used as query. 7. Kinase domain masked; amino acids 150-516 used as query. 8. Kinase domain masked; amino acids 1-100 used as query. 9. Kinase domain masked; amino acids 1-890 used as query. Asterisks indicate genes that appear elsewhere in the same table (in a different pathway). Click here for file

Additional file 10 **Selected developmental process genes identified in the *P. hawaiensis *transcriptome**. Hit ID indicates if gene hits were found assembled reads (A) or singletons (S). Sequence length (range) indicates the shortest and longest A or S hit sequences for each gene. Groups of hits of a given colour indicate transcriptome sequences that mapped to the same overlapping region of the BLAST target; hits of different colours indicate transcriptome sequences that map to different, non-overlapping regions of the BLAST target. Query organism was *D. melanogaster *for all cases. **Boldface **indicates genes also present in other tables (Additional Files 9, 11); asterisks indicate genes that appear elsewhere in the same table (in a different functional category). Click here for file

Additional file 11 **Selected genes involved in gametogenesis identified in the *P. hawaiensis *transcriptome**. Hit ID indicates if gene hits were found assembled reads (A) or singletons (S). Sequence length (range) indicates the shortest and longest A or S hit sequences for each gene. Groups of hits of a given colour indicate transcriptome sequences that mapped to the same overlapping region of the BLAST target; hits of different colours indicate transcriptome sequences that map to different, non-overlapping regions of the BLAST target. Query organism was *D. melanogaster *for all cases. Query sequence details: 1. S/T kinase domain was masked. 2. Dead box/Zn finger domains were masked. 3. HLH domain was masked 4. Peptidase C14 domain was masked. 5. Kinase domain masked; amino acids 175-372 used as query. 6. BTB domain used as query. 7. Kinase domain masked; amino acids 1-890 used as query. **Boldface **indicates genes also present in other tables (Additional Files 9, 10); asterisks indicate that genes are also present elsewhere (in a different functional category) in the same table. Click here for file

Additional file 12 **Representative of consecutively numbered isotigs with highly similar lengths**. An example of two isotigs which both have Cyclin D as their top BLAST hit (see Additional File 9), differ in length by only two nucleotides, and have highly similar sequences. Isotig07129 is 4,279 bp long; isotig07130 is 4,277 bp long. Only a portion of the sequence of each isotig is shown. Nucleotide positions differing between the two are indicated in black (likely to be SNPs), white (deletions) or grey (apparent sequence difference may be due to poor quality sequence (lower case letters) at this position). Click here for file
