# Supplementary material for: De novo assembly and characterization of a maternal and developmental transcriptome for the emerging model crustacean Parhyale hawaiensis
Source: BMC Genomics. 2011 Nov 25;12:581. doi: 10.1186/1471-2164-12-581 (PMC3282834; doi:10.1186/1471-2164-12-581)
Supplement: Additional file 1 — Embryonic stages pooled for creation of the P. hawaiensis transcriptome. Staging as per [55]. [file 1471-2164-12-581-S1.PDF]

Additional File 1

Stage distribution of embryos collected for preparation of cDNA.

Stage of embryos	Number of embryos
**1**	51
**2**	41
**3**	15
**4**	22
**5**	16
**6**	21
**7**	42
**8**	22
**9**	8
**10**	15
**11**	13
**12**	16
**13**	11
**14**	30
**15**	5
**16**	4
**17**	5
**18**	6
**19**	4
**20**	28
**21**	19
**22**	15
**23**	10
**24**	17
**25**	12
**26**	10
**27**	13
**Total**	**471**
