# Supplementary figures and images for: De novo assembly and characterization of a maternal and developmental transcriptome for the emerging model crustacean Parhyale hawaiensis
Source: BMC Genomics. 2011 Nov 25;12:581. doi: 10.1186/1471-2164-12-581 (PMC3282834; doi:10.1186/1471-2164-12-581)

Additional File 2

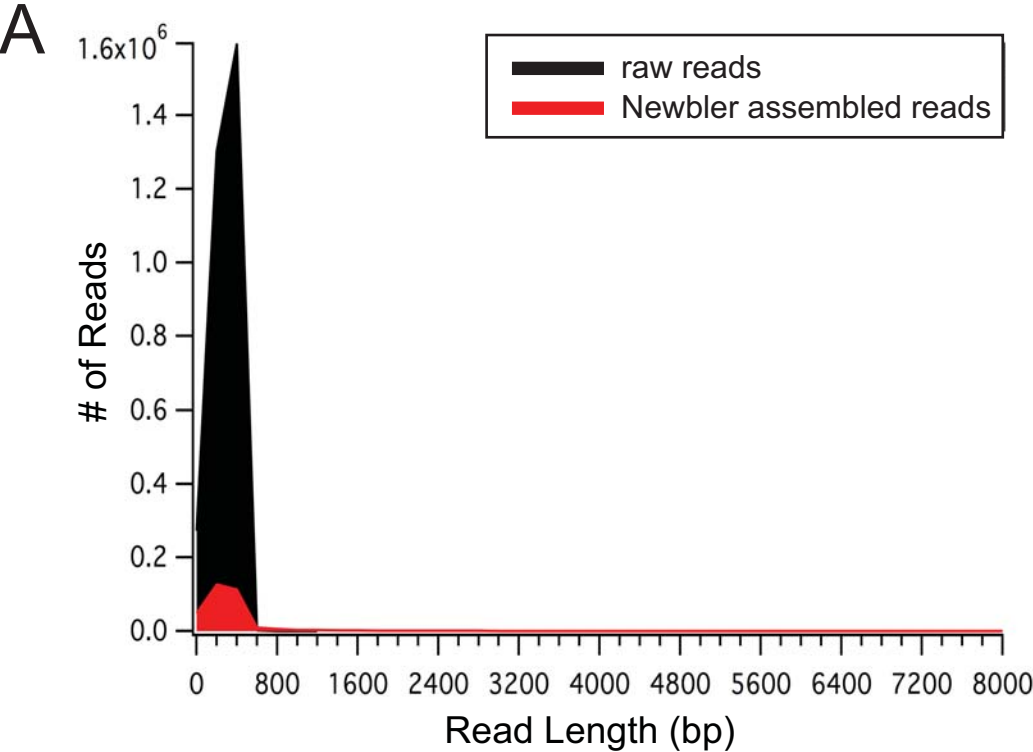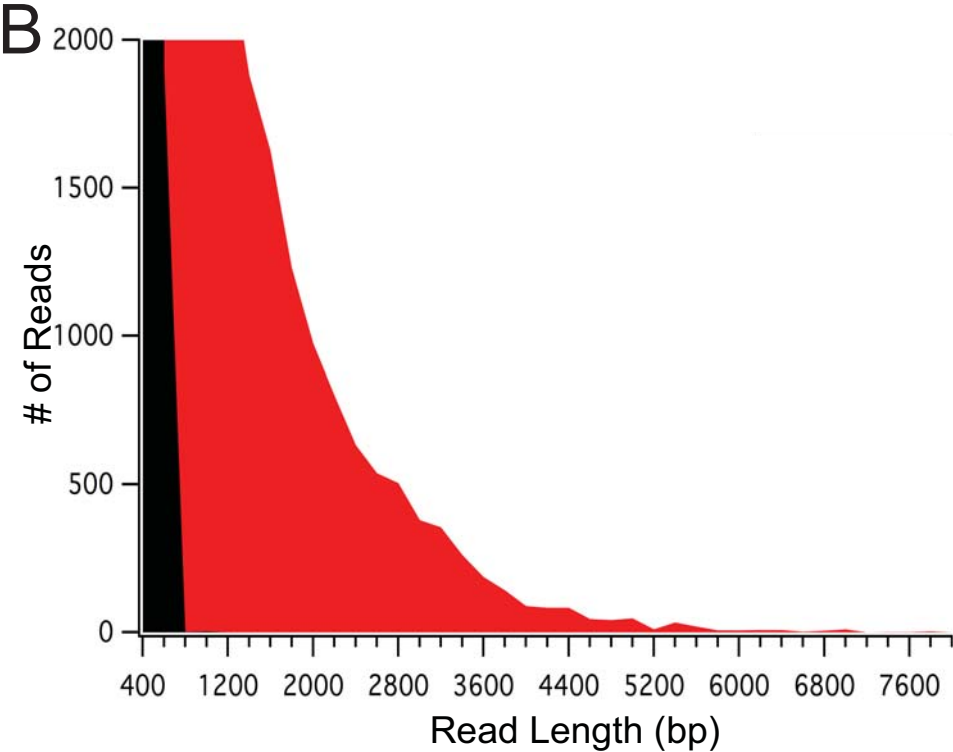

Supplement: Additional file 2 — Comparison of read lengths from Newbler v2.5 de novo assembly of the P. hawaiensis transcriptome. (A) Distribution of read lengths after assembly with Newbler v2.5 (red). (B) Distribution of read lengths of the shortest assembled reads and raw reads. The assembly yielded assembled reads of over ~4000 bp. [file 1471-2164-12-581-S2.PDF]
