# Supplementary material for: De novo assembly and characterization of a maternal and developmental transcriptome for the emerging model crustacean Parhyale hawaiensis
Source: BMC Genomics. 2011 Nov 25;12:581. doi: 10.1186/1471-2164-12-581 (PMC3282834; doi:10.1186/1471-2164-12-581)
Supplement: Additional file 4 — Analysis of the effect of trans-splicing transcripts on de novo transcriptome assembly. Assembly of all trimmed sequences compared to assembly of sequences lacking the trans-splicing leader sequences [47]. Number of BLAST hits reflects a search against the nr database with an E-value cut-off value of 1e-10. [file 1471-2164-12-581-S4.PDF]

Additional File 4

The effect of removing trans-spliced transcripts on *de novo* assembly of the *P. hawaiensis* transcriptome.

	**All reads**	**Only reads without trans-splicing leader sequence**
Assembled reads	3,157,373	3,154,789
Isotigs	35,301	35,529
Isotig N50	1,510	1,523
Mean # contigs per isotig	2.1	2.1
Contigs (“exons”)	89,664	85,574
Isogroups (“genes”)	25,735	25,536
Mean # isotigs per isogroup	1.4	1.4
Singletons (% of assembled reads)	276,564 (8.76%)	275,935 (8.75%)
# Unique BLAST hits	19,067	18,843
