# Supplementary material for: De novo assembly and characterization of a maternal and developmental transcriptome for the emerging model crustacean Parhyale hawaiensis
Source: BMC Genomics. 2011 Nov 25;12:581. doi: 10.1186/1471-2164-12-581 (PMC3282834; doi:10.1186/1471-2164-12-581)

Additional File 5

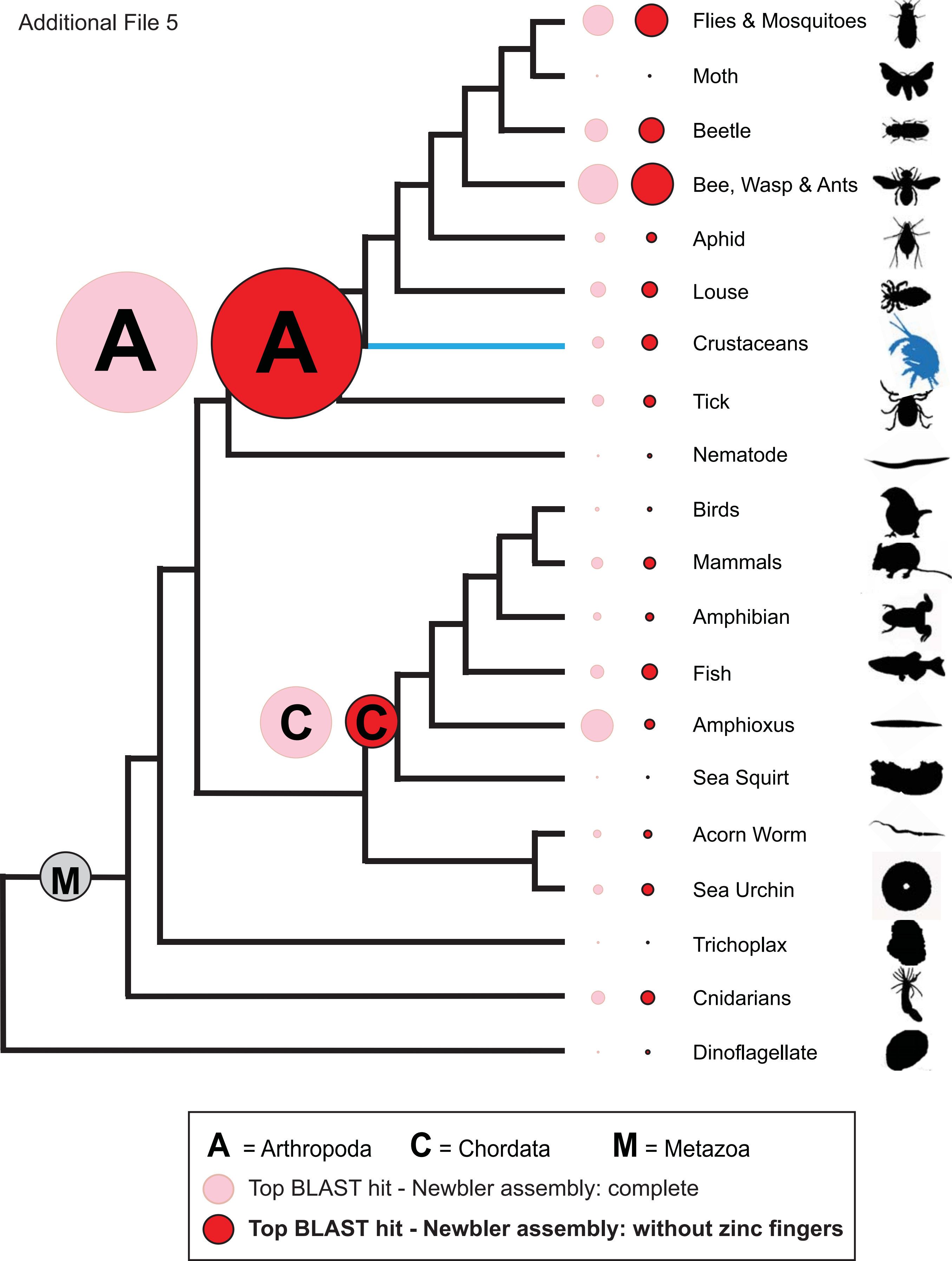

Supplement: Additional file 5 — Phylogenetic distribution of species of top unique BLAST hit for Newbler v2.5 assembly of the P. hawaiensis transcriptome. Of the unique BLAST hits to all non-redundant assembly products (isotigs + singletons), 90% were from species belonging to the clades shown. Over 50% of these top BLAST hits are from arthropod species. The large number (12.2%) of top BLAST hits in the complete assembly to sequences from Branchiostoma floridae is due to the high similarity of C2H2 zinc finger domain-containing sequences with a particular linker sequence (TGEKP) that is also highly represented in the genome of the aphid Acyrthosiphon pisum [48]. Red: values after removal of reads and sequences containing this domain. Phylogenetic tree modified from [63,74,75]. Hits from the following most abundant species are represented: D. mojavensis, D. willistoni, D. ananassae, D. grimshawi, D. pseudoobscura pseudoobscura, Aedes aegypti, Anopheles gambiae, Culex quinquefasciatus (Flies & Mosquitoes), Bombyx mori (Moth), Tribolium castaneum (Beetle), Harpegnathos saltator, Camponotus floridanus, Apis mellifera, Nasonia vitripennnis (Bee, Wasp & Ants), Acyrthosiphon pisum (Aphid), Pediculus humanus corporis (Louse), Ixodes scapularis (Tick), Penaeus monodon, Lepeophtheirus salmonis, Litopenaeus vannamei (Crustaceans), Caenorhabditis remanei (Nematode), Gallus gallus, Taeniopygia guttata (Birds), Rattus norvegicus, Mus musculus, Monodelphis domestica (Mammals), Xenopus laevis, X. tropicalis (Amphibian), Danio rerio, Tetraodon nigroviridis (Fish), Branchiostoma floridae (Amphioxus), Ciona intestinalis (Sea Squirt), Saccoglossus kowalevskii (Acorn Worm), Strongylocentrotus purpuratus (Sea Urchin), Trichoplax adherens (Trichoplax), Hydra magnipapillata, Nematostella vectensis (Cnidarians), Perkinsus marinus (Dinoflagellate). [file 1471-2164-12-581-S5.PDF]
