# Supplementary material for: De novo assembly and characterization of a maternal and developmental transcriptome for the emerging model crustacean Parhyale hawaiensis
Source: BMC Genomics. 2011 Nov 25;12:581. doi: 10.1186/1471-2164-12-581 (PMC3282834; doi:10.1186/1471-2164-12-581)
Supplement: Additional file 6 — Sequences with strong similarity to Daphnia pulex gene sequences identified in the de novo P. hawaiensis transcriptome. Because the D. pulex genome and nr are databases of inevitably different sizes, E-values shown here are for information only and are not strictly comparable. See text for additional details. [file 1471-2164-12-581-S6.PDF]

Additional File 6

Sequences with strong similarity to *Daphnia pulex* gene sequences identified in the *de novo P. hawaiensis* transcriptome

***P. hawaiensis* transcriptome sequence name**	NR BLAST				*D. pulex* BLAST	**(NR e-value) ÷ (*D.pulex* e-value)**
	e-value	*top hit species*	*Species Clade*	*description*	e-value	
GIAFTRM02H5QYS	0.000164683	*Acyrthosiphon pisum*	arthropod	star protein	6.44E-12	**3.E+07**
isotig15596	0.000144942	*Acyrthosiphon pisum*	arthropod	PREDICTED: similar to reverse transcriptase	3.25E-12	**4.E+07**
isotig15595	0.00223631	*Acyrthosiphon pisum*	arthropod	PREDICTED: similar to pol-like protein	3.06E-17	**7.E+13**
GIB53OK01C9MG7	0.00178957	*Pediculus humanus corporis*	arthropod	triacylglycerol lipase, pancreatic, putative	3.79E-13	**5.E+09**
GIAFTRM01AOYPP	0.000213202	*Pediculus humanus corporis*	arthropod	triacylglycerol lipase, pancreatic, putative	1.53E-16	**1.E+12**
GIB53OK01DZMOG	0.000216004	*Pediculus humanus corporis*	arthropod	triacylglycerol lipase, pancreatic, putative	1.48E-16	**1.E+12**
GIAFTRM01EUPF7	0.000211395	*Pediculus humanus corporis*	arthropod	triacylglycerol lipase, pancreatic, putative	1.26E-16	**2.E+12**
isotig10612	0.0360572	*Aedes aegypti*	arthropod	hypothetical protein	9.83E-11	**4.E+08**
isotig10613	0.0355852	*Aedes aegypti*	arthropod	hypothetical protein	9.72E-11	**4.E+08**
GIB53OK01BEIG6	1.1072	*Drosophila pseudoobscura pseudoobscura*	arthropod	GA12165	8.84E-11	**1.E+10**
GIB53OK01C9MM5	0.000165918	*Tribolium castaneum*	arthropod	hypothetical protein	7.13E-11	**2.E+06**
GIAFTRM01D95E6	0.000204485	*Drosophila persimilis*	arthropod	GL22267	5.27E-11	**4.E+06**
GAP9EXG06GW68F	0.000117688	*Nasonia vitripennis*	arthropod	PREDICTED: similar to CG1744-PA	3.63E-13	**3.E+08**
GIAFTRM02GLO7Y	0.000212279	*Pediculus humanus corporis*	arthropod	triacylglycerol lipase, pancreatic, putative	8.84E-15	**2.E+10**
GAP9EXG05F1T1C	0.0259067	*Drosophila teissieri*	arthropod	unknown protein	1.72E-17	**2.E+15**
contig32333	0.0339617	*Drosophila mojavensis*	arthropod	GI24390	2.07E-19	**2.E+17**
isotig14754	0.14676	*Ixodes scapularis*	arthropod	hypothetical protein	3.97E-23	**4.E+21**
isotig12959	0.31147	*Ixodes scapularis*	arthropod	hypothetical protein	2.43E-23	**1.E+22**
isotig14755	0.00668069	*Ixodes scapularis*	arthropod	hypothetical protein	1.36E-27	**5.E+24**

isotig12958	0.37496	*Ixodes scapularis*	arthropod	hypothetical protein	2.11E-26	**2.E+25**
GIAFTRM01BJRPD	0.000123529	*Mus musculus*	mammal	tudor domain containing 1	4.24E-11	**3.E+06**
GIB53OK02HQIDV	0.00237788	*Ornithorhynchus anatinus*	mammal	PREDICTED: similar to regeneration associated muscle protease	1.23E-26	**2.E+23**
GIAFTRM01D8IZA	0.0449664	*Tetraodon nigroviridis*	fish	unnamed protein product	1.33E-13	**3.E+11**
GAP9EXG05GC025	0.486607	*Gallus gallus*	bird	PREDICTED: hypothetical protein	1.61E-15	**3.E+14**
GIB53OK01DHBX9	0.000123896	*Nematostella vectensis*	cnidarian	hypothetical protein	7.60E-11	**2.E+06**
isotig27688	0.000684825	*Hydra magnipapillata*	cnidarian	PREDICTED: similar to predicted protein	2.67E-14	**3.E+10**
GIAFTRM02GS7JS	0.285627	*Oryza sativa*	plant	hypothetical protein	7.47E-11	**4.E+09**
GAP9EXG06G35K4	0.220332	*Sclerotinia sclerotiorum*	fungus	hypothetical protein	9.92E-12	**2.E+10**
GIB53OK02FPXMS	0.000279552	*Pseudomonas mendocina ymp*	bacteria	cyanophycinase	2.91E-15	**1.E+11**
GIB53OK01B96AH	0.000628388	*Myxococcus xanthus DK 1622*	bacteria	hypothetical protein	6.86E-30	**9.E+25**
