# Supplementary material for: De novo assembly and characterization of a maternal and developmental transcriptome for the emerging model crustacean Parhyale hawaiensis
Source: BMC Genomics. 2011 Nov 25;12:581. doi: 10.1186/1471-2164-12-581 (PMC3282834; doi:10.1186/1471-2164-12-581)
Supplement: Additional file 7 — Presence of existing P. hawaiensis GenBank accessions in the de novo transcriptome. Sequences of P. hawaiensis developmental genes from GenBank were used as a query to BLAST the de novo transcriptome. Most genes with hits had several matches in the transcriptome, among both assembled reads and singletons. [file 1471-2164-12-581-S7.PDF]

Additional File 7

The presence of pre-existing *P. hawaiensis* GenBank sequences in the de *novo P. hawaiensis* transcriptome.

Gene Name	Accession #	Original Sequence Length	#bp added by transcriptome	# 5' nt added	# 3' nt added	splice variants identified in transcriptome	Transcriptome Read Name	Consensus Region	Query Location
collier	FN557064	1883	0	0	0	208	GIAFTRM02JZUZI	209-445	741-955
			0	0	0	291	GAP9EXG06HGHE0	1-90	455-544
			0	0	0	0	GIB53OK01AYMZT	full	
Delta	DQ917570	4700	0	0	0	0	contig01219	full	
			0	0	0	0	isotig27755	full	
			0	0	0	0	isotig22358	full	
			0	0	0	0	isotig16703	full	
			0	0	0	0	contig01217	full	
			0	0	0	0	GIB53OK02H7WBD	full	
Notch	DQ917572	8701	0	0	0	0	GIAFTRM02GIVPJ	full	
			0	0	0	0	GIAFTRM02I2MOE	full	
			0	0	0	0	GAP9EXG05GAASS	full	
			0	0	0	0	GIB53OK01CKXYD	full	
			0	0	0	0	GIB53OK01D7FNS	full	
			0	0	0	316	GAP9EXG06HJ3NO	1-117	1637-1813
			0	0	0	1631	isotig10517	1-97	8600-8701
			0	0	0	1641	isotig10516	1-97	8600-8701
nanos	EU289288	2057	0	0	0	0	contig17249	full	
			0	0	0	0	contig17254	full	
			0	0	0	0	contig17145	full	
			0	0	0	0	contig17174	full	
			0	0	0	0	contig17232	full	
			0	0	0	0	contig17146	full	
			0	0	0	0	contig17208	full	
			0	0	0	0	GIAFTRM01E0TTH	full	
			0	0	0	0	contig17151	full	
			0	0	0	0	GAP9EXG05FR768	full	

			0	0	0	0	GIAFTRM02FMF9V	full	
			0	0	0	0	contig17163	full	
			0	0	0	0	GIAFTRM02IR7M8	full	
			0	0	0	0	contig17147	full	
odd-skipped	DQ917573	1333	0	0	0	0	isotig19431	full	
optix	EU908055	3654	0	0	0	400	GAP9EXG05GBZ0N	169-240	3548-3619
Pax3/7	HM347085	5074	0	0	0	0	GIAFTRM01EJ1LN	full	
			0	0	0	0	GAP9EXG06G5UHA	full	
			0	0	0	190	GIAFTRM01COC2I	1-236	3639-3874
			0	0	0	156	GIB53OK02IESL9	157-485	4484-4815
propsero	HM191476	817	116	116	0	0	GAP9EXG06HFGHB	1-266	2-267
			0	0	0	0	GIB53OK01BHCBI	full	
			0	0	0	0	GIAFTRM02F9OMW	full	
			459	0	459	0	isotig24415	460-753	511-803
short gastrulation	HM191474	5068	0	0	0	0	GIB53OK02IE0SF	full	
			0	0	0	0	GIB53OK01A8TUX	full	
			0	0	0	0	GIB53OK02FKYII	full	
			0	0	0	100	GIAFTRM02F0XQ6	1-232	4145-4372
			0	0	0	39	GIB53OK01A5PU9	1-213, 252-410	4-215, 254-406
			0	0	0	61	GIAFTRM01C49QK	44-216	44-215
			0	0	0	317	GIB53OK01CPNZQ	105-211	4145-4251
			0	0	0	276	GAP9EXG05F4HQ7	78-164	4380-4466
			0	0	0	207	GIAFTRM01EP2KQ	208-294	4967-5053
Ultrabithorax	FJ628449	2557	0	0	0	0	GIAFTRM02G2YVB	full	
			0	0	0	72	GIAFTRM02FHH2Z	1-134, 167-479	741-1054, 1087-1218
			0	0	0	0	GIB53OK02J5R82	full	
			0	0	0	342	GIAFTRM02FNBAI	275-423	1087-1233
vasa	EU726766	2834	0	0	0	0	isotig27190	full	
			0	0	0	0	isotig28240	full	
