# Supplementary material for: De novo assembly and characterization of a maternal and developmental transcriptome for the emerging model crustacean Parhyale hawaiensis
Source: BMC Genomics. 2011 Nov 25;12:581. doi: 10.1186/1471-2164-12-581 (PMC3282834; doi:10.1186/1471-2164-12-581)
Supplement: Additional file 9 — Selected signaling pathway genes identified in the P. hawaiensis transcriptome. Hit ID indicates if gene hits were found assembled reads (A) or singletons (S). Sequence length (range) indicates the shortest and longest A or S hit sequences for each gene. These results are shown graphically in Figure 7. Groups of hits of a given colour indicate transcriptome sequences that mapped to the same overlapping region of the BLAST target; hits of different colours indicate transcriptome sequences that map to different, non-overlapping regions of the BLAST target. Query organisms: Dm = D. melanogaster; Dr = Danio rerio; Xt = Xenopus tropicalis. Query sequence details: 1. Kinase domain was masked. 2. FERM domain used as query. 3. Amino acids 500-833 (Dl/Ser domain) used as query. 4. Amino acids 1-250 (groucho/TLE domain) used as query. 5. Kinase domain masked; amino acids 420-1390 used as query. 6. Kinase domain masked; amino acids 175-372 used as query. 7. Kinase domain masked; amino acids 150-516 used as query. 8. Kinase domain masked; amino acids 1-100 used as query. 9. Kinase domain masked; amino acids 1-890 used as query. Asterisks indicate genes that appear elsewhere in the same table (in a different pathway). [file 1471-2164-12-581-S9.PDF]

Additional File 9

Selected signaling pathway genes identified in the *de novo P. hawaiensis* transcriptome.

Process	# Hits	Hit ID (A/S)	Length (range)	Query Organism	Query Gene	Transcriptome Sequence Name(s)
HEDGEHOG						
*CK1**	5	A	1650-3879	*Dr*	*Csnkla1*	isotig04171, isotig04172, isotig04173, isotig04174, isotig04175
	1	S	487	*Dm*	*gish*	GIB53OK01BQG2U
camp-dependent protein kinase	2	A	1102	*Dm*	*Pka-C1*	isotig19762
		A	2027	*Dm*	*Pka-C3*	contig19788
cubitus interruptus	1	A	2820	*Dm*	*ci*	isotig16929
Decapentaplegic	1	A	975	*Dm*	*dpp*	isotig20685
Fused	1	A	2579	*Dm*[1]	*fu*	isotig17006
*GSK-3β**	1	A	872	*Dm*[1]	*sgg*	isotig21888
Rab23	1	A	745	*Dm*	*Rab23*	contig42822
Suppressor of fused	5	A, S	406-3656	*Dm*	*Su(fu)*	isotig05585, isotig05586, isotig05587, isotig05588, GIB53OK01A0R8L
*Slim b**	2	A	716-742	*Dm*	*slmb*	isotig25540, isotig24692
*Wnt**	4	S	326-425	*Dm*	*Wnt2*	GIAFTRM02F20NM, GIAFTRM01E06E7, GIB53OK01D17SK, GIB53OK02F0W2C
JAK/STAT						
AKT	1	A	695	*Dm*	*Akt1*	isotig26091
Cb1	2	S	387-431	*Dm*	*Cbl*	GIAFTRM02F64V8, GIAFTRM02JC8Q6
*CBP**	10	A	1024-2053	*Dm*	*nej*	isotig01234, isotig01235, isotig01236, isotig01237, isotig01238, isotig01239, isotig01240, isotig06748, isotig06749, isotig06750
Cyclin D	4	A	2054-4279	*Dm*	*CycD*	isotig07129, isotig07130, isotig07131, isotig17312
*GRB**	1	A	1124	*Dm*	*drk*	isotig19629
JAK	1	S	314	*Dm*[2]	*hop*	GIAFTRM01CXLBC
PI3K	2	A	3871-3888	*Dm*	*Pi3K21B*	isotig07171, isotig07172
Pim-1	1	A	3482	*Xt*	*pim1*	isotig16816

*SHP2**	2	S	318-466	*Dm*	*csw*	GIB53OK01DRDCY, GIAFTRM02JG2C8
SOCS	1	A	2113	*Dm*	*Socs36E*	contig12642
*SOS**	1	A	772	*Dm*	*sos*	isotig23898
Spred	1	S	308	*Dm*	*Spred*	GIB53OK01CR70W
STAM	1	S	323	*Dm*	*St-am*	GIB530K02IPC6E
STAT	1	S	470	*Dm*	*Stat92E*	GAP9EXG05F34AM
NOTCH						
APH-1	1	A	3054	*Dm*	*aph-1*	isotig16863
CIR	4	A, S	412-796	*Dm*	*CG6843*	contig12757, GIB53OK01AP7EV, GIAFTRM02FYJ09, GIAFTRM01BWE7G
Delta	1	A	619	*Dm*[3]	*Dl*	isotig27755
Deltex	3	A	751-1651	*Dm*	*dx*	isotig18630, isotig24394, isotig17863
*disheveled**	1	S	482	*Dm*	*dsh*	GAP9EXG05FXW6T
fringe	4	A	3395-3399	*Dm*	*fng*	isotig05593, isotig05594, isotig05595, isotig05596
*Groucho**	1	S	300	*Dm*[4]	*gro*	GIB53OK02H5Y2D
*HATs**	10	A	1024-2053	*Dm*	*nej*	isotig01234, isotig01235, isotig01236, isotig01237, isotig01238, isotig01239, isotig01240, isotig06748, isotig06749, isotig06750
HDAC	3	A	528-2699	*Dm*	*Rpd3*	isotig16965, contig38358, contig38356
Hes1/5	5	A	1267-1535	*Dr*	*her6*	isotig18896, isotig01472, isotig01473, isotig01474, isotig01475
Nicastrin	1	A	2422	*Dm*	*nct*	contig00347
Notch	3	S	448-493	*Dm*	*N*	GIAFTRM02GBWY7, GAP9EXG05GAASS, GAP9EXG06HJ3NO
Presenilin	1	S	276	*Dm*	*Psn*	GIAFTRM02H10N7
PSE2	4	A	1993-1994	*Dm*	*pen-2*	isotig05736, isotig05737, isotig05738, isotig05739
SKIP	1	S	384	*Dm*	*Bx42*	GIAFTRM02GTTL4
Tace	1	S	471	*Dm*	*Tace*	GIAFTRM02HLATS
WNT						
Axam	1	S	415	*Dr*	*wu:fb78c01*	GIB53OK01CADPS
*beta-TrCP**	2	A	716-742	*Dm*	*slmb*	isotig25540, isotig24692
c-jun	1	A	1744	*Dr*	*jun*	isotig17712
CaMKII	4	A, S	258-716	*Dm*[7]	*CaMKII*	GIB53OK01B77B2, GIAFTRM02HFVOZ, GIB53OK02FVWCO, isotig14738
*CBP**	10	A	1024-2053	*Dm*	*nej*	isotig01234, isotig01235, isotig01236, isotig01237, isotig01238, isotig01239, isotig01240, isotig06748, isotig06749, isotig06750

*CK1 alpha**	5	A	1650-3879	*Dr*	*Csnkla1*	isotig04171, isotig04172, isotig04173, isotig04174, isotig04175
	1	S	487	*Dm*	*gish*	GIB53OK01BQG2U
CK1ε	1	A	880	*Dr*	*csnk1e*	isotig21772
CK2	11	A	434-2106	*Dm*	*Ss1*	isotig17268, contig22385
			1998	*Dm*	*CkIIbeta*	contig29314
			4354-4882	*Dm*	*CkIIalpha*	isotig01646, isotig01647, isotig01648, isotig01649, isotig01650, isotig01651, isotig01652, isotig01653
camp-dependent protein kinase	2	A	1102	*Dm*	*Pka-C1*	isotig19762
		A	2027	*Dm*	*Pka-C3*	contig19788
CaN	5	A	786-2304	*Dm*	*CanA1*	isotig23630, isotig19714
					CanB2	isotig11973, isotig11974
					elm	contig30094
*Cul1**	3	A	1947-5192	*Dm*	*lin19*	isotig10423, isotig10424, isotig11517
*disheveled**	1	S	482	*Dm*	*dsh*	GAP9EXG05FXW6T
Ebi1	1	S	338	*Dm*	*ebi*	GIAFTRM01COVFU
Frizzled	2	A, S	713<br>372	*Dm*<br>*Dm*	*fz*<br>*fz2*	isotig25634<br>GIB53OK01EXZZC
*GSK-3β**	1	A	872	*Dm*[1]	*sgg*	isotig21888
*Groucho**	1	S	300	*Dm*[4]	*gro*	GIB53OK02H5Y2D
JNK	1	A	5027	*Dm*[6]	*bsk*	isotig16745
NFAT	2	S	430-437	*Dm*	*NFAT*	GIAFTRM02GWQ5S, GIAFTRM01CFG05
PKC	3	S	256-429	*Dm*	*InaC*	GIAFTRM01DR8NT, GIB53OK01DQHQZ
					Pkc53E	GIB53OK01C3SXA
PLC	9	S	280-465	*Dm*	*norpA*	GIB53OK01BKSR4, GIB53OK02GTBUS, GIB53OK02GDILS, GIB53OK01EXM1Z, GIB53OK02JVGTA, GIB53OK01CO34L, GIB53OK02F95N0, GIAFTRM01EZD87, GIB53OK02H5GWA
*PP2A**	2	A, S	365-1844	*Dm*	*Pp2A-29B*	contig32429, GAP9EXG05FTITZ
	2	A	874-1143	*Dm*	*wdb*	isotig19487, isotig21842
	2	A, S	485-1855	*Dm*	*mts*	GIAFTRM01DK761, isotig22169
	3	A, S	444-526	*Dm*	*PP2A-B'*	isotig30375, GIAFTRM01ENRYI, GIB53OK01CHYGL
ProteinS2	4	A	731-2239	*Dm*	*pont*	isotig21936, contig44209, isotig27389, contig44204
*Rbxl**	10	A, S	364-856	*Dm*	*Roc1a*	contig37731, contig37732, GIAFTRM02GRGCP, GIAFTRM02F2KYT, GIAFTRM01BSBHO, GIAFTRM01D9IU7, isotig22059, GIB53OK02FSMUQ,

						GIB53OK02J3YXD, GIB53OK02JZQA6
*RhoA**	4	A	3279-3344	*Dm*	*Rho1*	isotig06399, isotig06400, isotig06401, isotig06402
*ROCK2**	1	S	473	*Dm*[5]	*rok*	GIAFTRM01A0RNJ
Siah-1	2	A	2317-3646	*Dm*	*sina*	isotig11603, isotig11604
SIP	1	A	906	*Dm*	*CG3226*	contig22122
*Skp1**	2	A	2531-2532	*Dm*	*SkpC*	isotig10265, isotig10266
SMAD3	1	S	468	*Dm*	*Smox*	GIAFTRM01BGPRW
Uterine	4	A	3514-3529	*Mm*	*Mmp7*	isotig04881, isotig04882, isotig04883, isotig04884
*Wnt**	4	S	326-425	*Dm*	*Wnt2*	GIAFTRM02F20NM, GIAFTRM01E06E7, GIB53OK01D17SK, GIB53OK02F0W2C
TGF-BETA						
ActivinRII	1	A	1586	*Dr*	*acvr2b*	contig13339
BMPRI	2	A, S	359-1407	*Dr*	*bmpr1ba*	isotig18455, GIAFTRM01C5G7U
*Cul1**	3	A	1947-5192	*Dm*	*lin19*	isotig10423, isotig10424, isotig11517
Decapentaplegic	1	A	975	*Dm*	*dpp*	isotig20685
*ERK**	1	A	728	*Dm*	*rl*	isotig25011
*p300**	10	A	1024-2053	*Dm*	*nej*	isotig01234, isotig01235, isotig01236, isotig01237, isotig01238, isotig01239, isotig01240, isotig06748, isotig06749, isotig06750
p70S6K	1	A	920	*Dm*	*S6K*	isotig21264
*Rbx1**	10	A, S	364-856	*Dm*	*Roc1a*	contig37731, contig37732, GIAFTRM02GRGCP, GIAFTRM02F2KYT, GIAFTRM01BSBHO, GIAFTRM01D9IU7, isotig22059, GIB53OK02FSMUQ, GIB53OK02J3YXD, GIB53OK02JZQA6
*RhoA**	4	A	3279-3344	*Dm*	*Rho1*	isotig06399, isotig06400, isotig06401, isotig06402
*ROCK1**	1	S	473	*Dm*[5]	*rok*	GIAFTRM01A0RNJ
*Skp1**	2	A	2531-2532	*Dm*	*skpc*	isotig10265m isotig10266
Smad1/5/8	1	S	288	*Dm*	*Mad*	GIB53OK01B2DPO
THBS1	1	A	484	*Dr*	*thbs4b*	GIAFTRM02GCU0H
*PP2A**	2	A, S	365-1844	*Dm*	*Pp2A-29B*	contig32429, GAP9EXG05FTITZ
	2	A	874-1143	*Dm*	*wdb*	isotig19487, isotig21842
	2	A, S	485-1855	*Dm*	*mts*	GIAFTRM01DK761, isotig22169
	3	A, S	444-526	*Dm*	*PP2A-B'*	isotig30375, GIAFTRM01ENRYI, GIB53OK01CHYGL
MAPK						
*Csw**	2	S	318-466	*Dm*	*csw*	GIB53OK01DRDCY, GIAFTRM02JG2C8

*Drk**	1	A	1124	*Dm*	*drk*	isotig19629
Dsor1	2	A	4331-4336	*Dm*[8]	*Dsor1*	isotig10107, isotig10108
Egfr	2	S	420-434	*Dm*[9]	*Egfr*	GIAFTRM01CLKO7, GIB53OK02HG2L2
Gap1	2	S	399-438	*Dm*	*Gap1*	GIAFTRM02JJTOU, GIAFTRM01CDCB7
Ras85D	2	A	2427	*Dm*	*Ras85D*	isotig10293, isotig10294
*Rolled**	1	A	728	*Dm*	*rl*	isotig25011
*Sos**	1	A	772	*Dm*	*sos*	isotig23898
Ts1	1	A	780	*Dm*	*tsl*	Isotig14953
Yan	1	A	691	*Dm*	*aop*	isotig26139
