# Supplementary material for: De novo assembly and characterization of a maternal and developmental transcriptome for the emerging model crustacean Parhyale hawaiensis
Source: BMC Genomics. 2011 Nov 25;12:581. doi: 10.1186/1471-2164-12-581 (PMC3282834; doi:10.1186/1471-2164-12-581)
Supplement: Additional file 10 — Selected developmental process genes identified in the P. hawaiensis transcriptome. Hit ID indicates if gene hits were found assembled reads (A) or singletons (S). Sequence length (range) indicates the shortest and longest A or S hit sequences for each gene. Groups of hits of a given colour indicate transcriptome sequences that mapped to the same overlapping region of the BLAST target; hits of different colours indicate transcriptome sequences that map to different, non-overlapping regions of the BLAST target. Query organism was D. melanogaster for all cases. Boldface indicates genes also present in other tables (Additional Files 9, 11); asterisks indicate genes that appear elsewhere in the same table (in a different functional category). [file 1471-2164-12-581-S10.PDF]

Additional File 10

Selected developmental process genes identified in the *de novo P. hawaiensis* transcriptome.

Process	# Hits	Hit ID (A/S)	Length (range)	Query Organism	Query Gene	Transcriptome Sequence Name(s)
MATERNAL GENES						
ANTERIOR GROUP						
bicoid interacting protein 1	1	A	464	*Dm*	*Binl*	contig28994
POSTERIOR GROUP						
Bruno	1	A	590	*Dm*	*aret*	isotig16204
Cyclin B	3	A	2035-2041	*Dm*	*CycB*	isotig08773, isotig08774, isotig08775
mago nashi	2	A	1230-1235	*Dm*	*mago*	isotig10774, isotig10775
nanos	1	A	1048	*Dm*	*nos*	contig17249
pumilio	6	S	299-406	*Dm*	*pum*	GIAFTRM01DD2ST, GIB53OK02FM8FO, GIB53OK01C502X, GIAFTRM01EM7Q9, GIAFTRM02GXICC, GIAFTRM02GHD5G
vasa	1	A	647	*Dm*	*vas*	isotig27190
TERMINAL GROUP						
corkscrew	**2**	**S**	**318-466**	***Dm***	***csw***	**GIB53OK01DRDCY, GIAFTRMO2JG2C8**
dead ringer	1	A	732	*Dm*	*retn*	isotig24971
huckebein	1	S	380	*Dm*	*hkb*	GIB53OK01EBW1Y
Ras oncogene at 85D	**2**	**A**	**2427**	***Dm***	***Ras85D***	**isotig10293, isotig10294**
rolled	**1**	**A**	**728**	***Dm***	***rl***	**isotig25011**
torso-like	**1**	**A**	**780**	***Dm***	***tsl***	**isotig14953**
DORSAL GROUP						
cactus	1	A	446	*Dm*	*cact*	isotig34140
cornichon	4	A	1304-1876	*Dm*	*cni*	isotig05798, isotig05799, isotig05800, isotig05801
**DER/torpedo**	**2**	**S**	**420-434**	***Dm*** [9]	***Egfr***	**GIAFTRM01CLKO7**
pelle	2	A	1677-2731	*Dm*	*pll*	isotig11853, isotig11854
pipe	2	A	1787-2673	*Dm*	*pip*	isotig08940, isotig08941

rhomboid	1	A	1333	*Dm*	*rho*	isotig18676
snake	1	S	458	*Dm*	*snk*	GIB53OK01CAW2Y
squid	1	A	1076	*Dm*	*spd*	isotig19932
toll	3	A	988-1553	*Dm*	*TL*	isotig20600, isotig19162, isotig18073
windbeutel	1	A	794	*Dm*	*wbl*	isotig23269
ZYGOTICALLY TRANSCRIBED GENES						
abdominal A	2	S	492-536	*Dm*	*abd-A*	GIAFTRM01C7EEF, GIAFTRM01A1BYJ
Deformed	2	S	319-327	*Dm*	*Dfd*	GIB53OK02FVI7X, GAP9EXG06GZ0F6
labial	1	S	276	*Dm*	*lab*	GIAFTRM02JX0K6
**Ultrabithorax**	**1**	**S**	**518**	***Dm***	***Ubx***	**GIAFTRM02FHH2Z**
GAP, PAIR RULE, AND RELATED GENES						
GAP GENES						
cap n collar	2	S	357-376	*Dm*	*cnc*	GIB53OK01DY8IY, GIB53OK01APPLB
caudal	1	A	1747	*Dm*	*cad*	isotig17704
crocodile	2	A	418-436	*Dm*	*croc*	isotig16147, isotig16148
empty spiracles	1	A	942	*Dm*	*ems*	isotig20933
**huckebein**	**1**	**S**	**380**	**Dm**	***hkb***	**GIB53OK01EBWIY**
**hunchback**	**1**	**A**	**1298**	**Dm**	***hb***	**isotig18835**
knirps	1	A	753	*Dm*	*kni*	isotig24470
**knot**	**1**	**S**	**455**	**Dm**	***knot***	**GIAFTRM02JZUZI**
krupple	1	A	3269	*Dm*	*kr*	isotig11951
ocelliless	1	A	481	*Dm*	*otd*	isotig32018
sloppy paired 2	1	A	626	*Dm*	*slp2*	isotig27628
PAIR RULE GENES						
even skipped	1	A	1616	*Dm*	*eve*	isotig17948
odd paired	3	A	421-1282	*Dm*	*opa*	contig33284, isotig14898, contig33280
odd skipped	6	A	1161-2870	*Dm*	*odd*	isotig17496, isotig16912, isotig19431, isotig06114, isotig06115, isotig06116,
runt	2	A	3397-3461	*Dm*	*run*	isotig10142, isotig10141
**sloppy paired 2**	**1**	**A**	**626**	**Dm**	***slp2***	**isotig27628**
Tenascin major	1	S	483	*Dm*	*Ten-m*	GIAFTRM02HPJBX
GENES INVOLVED IN REGULATION OF GAP AND PAIR RULE GENES						
Chip	1	S	329	*Dm*	*chi*	GAP9EXG06G5UJD

**dead ringer**	**1**	**A**	**732**	**Dm**	***retn***	**isotig24971**
eyelid	1	S	322	*Dm*	*osa*	GIB53OK02IQUGI
ftz transcription factor 1	1	S	365	*Dm*	*ftz-f1*	GAP9EXG06GU4JI
**hopscotch**	**1**	**S**	**314**	**Dm[2]**	***hop***	**GIAFTRM01CXLBC**
**marelle**	**1**	**S**	**470**	**Dm**	***Stat92E***	**GAP9EXG05F34AM**
**Rpd3**	**3**	**A**	**528-2699**	**Dm**	***Rpd3***	**isotig16965, contig38358, contig38356**
Sir2	2	A	1870-2861	*Dm*	*Sir2*	isotig11782, isotig11781
**squid**	**1**	**A**	**1076**	**Dm**	***spd***	**isotig19932**
