# Supplementary material for: De novo assembly and characterization of a maternal and developmental transcriptome for the emerging model crustacean Parhyale hawaiensis
Source: BMC Genomics. 2011 Nov 25;12:581. doi: 10.1186/1471-2164-12-581 (PMC3282834; doi:10.1186/1471-2164-12-581)
Supplement: Additional file 11 — Selected genes involved in gametogenesis identified in the P. hawaiensis transcriptome. Hit ID indicates if gene hits were found assembled reads (A) or singletons (S). Sequence length (range) indicates the shortest and longest A or S hit sequences for each gene. Groups of hits of a given colour indicate transcriptome sequences that mapped to the same overlapping region of the BLAST target; hits of different colours indicate transcriptome sequences that map to different, non-overlapping regions of the BLAST target. Query organism was D. melanogaster for all cases. Query sequence details: 1. S/T kinase domain was masked. 2. Dead box/Zn finger domains were masked. 3. HLH domain was masked 4. Peptidase C14 domain was masked. 5. Kinase domain masked; amino acids 175-372 used as query. 6. BTB domain used as query. 7. Kinase domain masked; amino acids 1-890 used as query. Boldface indicates genes also present in other tables (Additional Files 9, 10); asterisks indicate that genes are also present elsewhere (in a different functional category) in the same table. [file 1471-2164-12-581-S11.PDF]

Additional File 11

Selected gametogenesis genes identified in the *de novo P. hawaiensis* transcriptome (Newbler v2.5 assembly).

Process	# Hits	Hit ID (A/S)	Length (range)	*Query Organism*	Query Gene	Transcriptome Sequence Names
SPERMATOGENESIS						
TRANSCRIPTION FACTOR						
Enchancer of bithorax	3	S	411-452	*Dm*	*E(bx)*	GIAFTRM01A4P7F, GIAFTRM01CPPM3, GIAFTRM01EC51W
eyes absent	1	S	486	*Dm*	*eya*	GIAFTRM01BAZXY
*Heat shock factor**	1	A	2157	*Dm*	*Hsf*	isotig06696
MBD-like	1	A	1040	*Dm*	*MBD-like*	contig35950
*Myb oncogene-like**	1	A	797	*Dm*	*Myb*	isotig23283
sine oculis	1	S	403	*Dm*	*so*	GAP9EXG05F77JI
*TATA box binding protein-related factor 2**	1	A	1030	*Dm*	*Tfr2*	isotig20178
vismay	1	S	401	*Dm*	*vis*	GIAFTRM01DKB1Z
CYTOSKELETON						
*abnormal spindle**	1	S	473	*Dm*	*asp*	GAP9EXG05F227G
beta 2 Tubulin	2	A	479	*Dm*	*βTub85D*	isotig07475, isotig07476
*beta1 tubulin**	7	A	1522-3151	*Dm*	*βTub56D*	isotig04869, isotig04870, isotig04871, isotig04872, isotig04873, isotig04874, isotig18155
*chickadee**	3	A	1018-5120	*Dm*	*chic*	isotig11514, isotig11375, isotig11513
*diaphanous**	1	S	284	*Dm*	*dia*	GIAFTRM01C7J9C
Kinesin like protein at 61F	1	S	458	*Dm*	*Klp61F*	GIAFTRM02FUJ93
peanut	1	A		*Dm*	*pnut*	isotig32706
*Rac1**	5	A	2613-4872	*Dm*	*Rac1*	isotig02074, isotig02075, isotig02076, isotig02077, isotig02078
*Spectrin 1**	7	A, S	256-1440	*Dm*	*alpha-Spec*	isotig18382, GIAFTRM02F0YPD, GIAFTRM02JWGUW, GIB53OK01AI3OT, GIAFTRM01B0FL5, GIB53OK02IV02I, GIB53OK02I37MA
*twinstar**	2	A	387-434	*Dm*	*tsr*	contig26952, isotig34793

*zipper**	4	S	423-524	*Dm*	*zip*	GIAFTRM02JY0HB, GIAFTRM01AXRAK, GIAFTRM01ELKZE, GIAFTRM01ENSHU
OTHER PROCESSES IN SPERMATOGENESIS						
arrest	**1**	**A**	**590**	***Dm***	***aret***	**isotig16204**
*aubergine**	2	A, S	458-489	*Dm*	*aub*	isotig33798, GIB53OK01DBEHU
boule	1	S	2156-2161	*Dm*	*bol*	GIAFTRM02GOTOR
*Btk family kinase at 29A**	5	S	402-346	*Dm*	*Btk29A*	GIB53OK02FHDVN, GIAFTRM01CKL2U, GIB53OK01EDQ85, GIAFTRM02HZF7J, GIAFTRM01A2C1B
Calmodulin	1	A	2700	*Dm*	*cam*	contig39461
*capsuleen**	2	S	412-463	*Dm*	*csu1*	GIAFTRM02G9BFL, GIB53OK02IE8IK
cdc2	3	A	1981-4352	*Dm*	*cdc2*	isotig07135, isotig07136, isotig07137
courtless	1	A	1483	*Dm*	*crl*	isotig18238
Cyclin A	1	A	1516	*Dm*	*CycA*	contig28480
Cyclin B	**3**	**A**	**2035-2041**	***Dm***	***CycB***	**isotig08773, isotig08774, isotig08775**
Cytochrome c proximal	1	A	695	*Dm*	*Cyt-c-p*	isotig26100
Cytochrome c distal	1	A	818	*Dm*	*Ctc-c-d*	isotig22668
Dynamin related protein 1	2	A	804-3166	*Dm*	*Drp1*	isotig23218, isotig16850
*effete**	3	A, S	391-2242	*Dm*	*eff*	isotig02238, isotig02239, GIB53OK01DP89J
Fmr1	1	A	777	*Dm*	*Fmr1*	isotig23785
Gilgamesh	**1**	**S**	**487**	***Dm***	***gish***	**GIB53OK01BQG2U**
*hephaestus**	1	A	481	*Dm*	*heph*	isotig32718
*Ice**	1	S	453	*Dm*[4]	*Ice*	GIAFTRM01EEGBL
Karyopherin a1	5	A, S	243-598	*Dm*	*Kap-a1*	contig48144, GIB53OK01CN6WI, GIB53OK02J5PXC, GIB53OK01EQJKN, GIAFTRM02G1JIO
*loquacious**	3	A	1265-1763	*Dm*	*loqs*	isotig17669, isotig12737, isotig12738
no receptor potential	**9**	**S**	**280-465**	***Dm***	***norpA***	**GIB53OK01BKSR4, GIB53OK02GTBUS, GIB53OK02GDILS, GIB53OK01EXM1Z, GIB53OK02JVGTA, GIB53OK01CO34L, GIB53OK02F95N0, GIAFTRM01EZD87, GIB53OK02H5GWA**
*Nucleolar protein at 60B**	1	A	1626	*Dm*	*Nop60B*	isotig17924
parkin	1	A	2107	*Dm*	*park*	isotig17265
pavarotti	2	S	462-468	*Dm*	*par*	GIAFTRM02IO385, GIAFTRM02IPPSA
*pelota**	1	A	1339	*Dm*	*pelo*	isotig18683
*Rab-protein 11**	1	A	945	*Dm*	*Rab11*	isotig20992
Rheb	1	A	2415	*Dm*	*Rheb*	isotig17081

rhomboid 7	2	A	787-796	*Dm*	*rho-7*	contig38394, contig38395
*Stem cell tumor/rhomboid-2**	3	A	2007-2166	*Dm*	*Stet*	isotig08759, isotig08760, isotig08761
string	1	A	636	*Dm*	*stg*	isotig27401
Syntaxin 5	3	A	626-1850	*Dm*	*syx5*	contig29998, contig30000, contig29983
glass bottom boat	1	S	494	*Dm*	*gbb*	GIAFTRM01B7BBJ
thick veins	**2**	**A, S**	**359-1407**	***Dr***	***bmpr1ba***	**isotig18455, GIAFTRM01C5G7U**
vav	1	A	1047	*Dm*	*vav*	isotig20094
OOGENESIS						
MAINTENANCE AND DIVISION OF GERM LINE STEM CELLS						
decapentaplegic	**1**	**A**	**975**	***Dm***	***dpp***	**isotig20685**
Dicer	1	A	1236	*Dm*	*Dcr-1*	isotig19090
dishevelled	**1**	**S**	**482**	***Dm***	***dsh***	**GAP9EXG05FXW6T**
*effete**	3	A, S	391-2242	*Dm*	*eff*	isotig02238, isotig02239, GIB53OK01DP89J
fused	**1**	**A**	**2579**	***Dm***[1]	***fu***	**isotig17006**
*loquacious**	3	A	1265-1763	*Dm*	*loqs*	isotig17669, isotig12737, isotig12738
nanos	**1**	**A**	**1048**	***Dm***	***nos***	**contig17249**
*ovo**	5	A, S	425-1541	*Dm*	*ovo*	GIB53OK02F9CZ7, GIAFTRM01C6X8Y, GIB53OK02F3ANY, GIAFTRM02IUB9D, isotig18095
*pelota**	1	A	1339	*Dm*	*pelo*	isotig18683
pumilio	**6**	**S**	**299-406**	***Dm***	***pum***	**GIAFTRM01DD2ST, GIB53OK02FM8FO, GIB53OK01C502X, GIAFTRM01EM7Q9, GIAFTRM02GXICC, GIAFTRM02GHD5G**
*sans fille**	4	A	289-941	*Dm*	*snf*	isotig13572, isotig13573, contig63129, contig63131
shaggy	**1**	**A**	**872**	***Dm***[1]	***sgg***	**isotig21888**
vasa	**1**	**A**	**647**	***Dm***	***vas***	**isotig27190**
OOCYTE DETERMINATION AND FORMATION OF AP AXIS						
4EHP	4	A	1299-1305	*Dm*	*4EHP*	isotig05881, isotig05882, isotig05883, isotig05884
*alpha Spectrin**	7	A, S	256-1440	*Dm*	*alpha-Spec*	isotig18382, GIAFTRM02F0YPD, GIAFTRM02JWGUW, GIB53OK01AI3OT, GIAFTRM01B0FL5, GIB53OK02IV02I, GIB53OK02I37MA
atypical protein kinase C	**1**	**A**	**256**	***Dm***	**Pkc53E**	**GIB53OK01C3SXA**
*beta1 tubulin**	7	A	1522-3151	*Dm*	*betaTub 56D*	isotig04869, isotig04870, isotig04871, isotig04872, isotig04873, isotig04874, isotig18155
*Bicaudal D**	3	S	341-446	*Dm*	*BicD*	GIAFTRM01A27KC, GIB53OK02F7E1Q, GIB53OK02HFKM7

cAMP-dependent protein kinase 1*	**1**	**A**	**1102**	***Dm***	***Pka-C1***	**isotig19762**
collier	1	S	455	*Dm*	*knot*	GIAFTRM02JZUZI
*COP9 complex homolog subunit 5**	1	A	935	*Dm*	*CSN5*	contig35647
Dynein heavy chain 64C	3	A, S	377-2270	*Dm*	*Dhc64c*	GIAFTRM02GEPAB, GIB53OK01BTS9Z, isotig08869
egalitarian	1	S	457	*Dm*	*egl*	GIB53OK01EKEVI
egghead	3	S	380-468	*Dm*	*egh*	GIAFTRM02F0HWW, GIAFTRM01C8ZKU, GIAFTRM01C9MZJ
gamma tubulin at 37C	1	A	2033	*Dm*	*gamma Tub37C*	isotig17332
Helicase at 25E	1	S	327	*Dm*	*Hel25E*	GIAFTRM02GXX4S
hu-li tai shao	2	S	432-436	*Dm*	*hts*	GIAFTRM02H3WPB, GIAFTRM02HLYJJ
Kinesin heavy chain	2	S	498-509	*Dm*	*khc*	GIB53OK01ECOW7, GIAFTRM01DH6Z1
*licorne**	1	A	742	*Dm*	*lic*	isotig24724
lkb1	1	A	1407	*Dm*	*lkb1*	isotig14335
*okra**	1	A	1035	*Dm*	*okr*	isotig20208
*ovo**	5	A, S	425-1541	*Dm*	*ovo*	GIB53OK02F9CZ7, GIAFTRM01C6X8Y, GIB53OK02F3ANY, GIAFTRM02IUB9D, isotig18095
par-6	1	A	341-1365	*Dm*	*par-6*	contig30838, contig30820, contig30819
pumilio	**6**	**S**	**299-406**	***Dm***	***pum***	**GIAFTRM01DD2ST, GIB53OK02FM8FO, GIB53OK01C502X, GIAFTRM01EM7Q9, GIAFTRM02GXICC, GIAFTRM02GHD5G**
Rab-protein 6	3	A	2592-2621	*Dm*	*Rab6*	isotig03322, isotig03323, isotig03324
*Stem cell tumor/rhomboid-2**	3	A	2007-2166	*Dm*	*Stet*	isotig08759, isotig08760, isotig08761
Ultrabithorax	1	S	518	*Dm*	*Ubx*	GIAFTRM02FHH2Z
FORMATION OF DV AXIS						
*COP9 complex homolog subunit 5**	1	A	935	*Dm*	*CSN5*	contig35647
cornichon	**5**	**A**	**1304-1876**	***Dm***	***cni***	**isotig05798, isotig05799, isotig05800, isotig05801**
*licorne**	1	A	742	*Dm*	*lic*	isotig24724
*okra**	1	A	1035	*Dm*	*okr*	isotig20208
pipe	**2**	**A**	**1787-2673**	***Dm***	***pip***	**isotig08940, isotig089841**
*squid**	1	A	1076	*Dm*	*sqd*	isotig19932
trailer hitch	1	A	1113	*Dm*	*tral*	isotig10860, isotig10861
ACTING EARLY IN FOLLICLE CELLS (DORSAL GROUP)						
*alpha Spectrin**	7	A, S	256-1440	*Dm*	*alpha-Spec*	isotig18382, GIAFTRM02F0YPD, GIAFTRM02JWGUW, GIB53OK01AI3OT, GIAFTRM01B0FL5, GIB53OK02IV02I,

						GIB53OK02I37MA
broad	1	A	562	*Dm*	*br*	contig08643
bunched	4	A, S	360-2977	*Dm*	*bun*	isotig11667, GIB53OK02H0LXA, isotig11668, GIAFTRM01D9IZJ
*chickadee**	3	A	1018-5120	*Dm*	*chic*	isotig11514, isotig11375, isotig11513
corkscrew	**2**	**S**	**318-466**	***Dm***	***csw***	**GIB53OK01DRDCY, GIAFTRMO2JG2C8**
Delta	**1**	**A**	**619**	***Dm***[3]	***Dl***	**isotig27755**
DER/torpedo	**2**	**S**	**420-434**	***Dm***[7]	***Egfr***	**GIAFTRM01CLKO7, GIB53OK02HG2L2**
dodo	2	A	399-435	*Dm*	*dod*	contig23139, contig23095
mago nashi	**2**	**A**	**1230-1235**	***Dm***	***mago***	**isotig10774, isotig10775**
neuralized	2	S	442-705	*Dm*	*neur*	GIB53OK01CF82S, isotig25761
Notch	**3**	**S**	**448-493**	***Dm***	***N***	**GIAFTRM02GBWY7, GAP9EXG05GAASS, GAP9EXG06HJ3NO**
pointed	1	S	523	*Dm*	*pnt*	GIAFTRM01BGKIP
*Rac1**	5	A	2613-4872	*Dm*	*Rac1*	isotig02074, isotig02075, isotig02076, isotig02077, isotig02078
Ras oncogene at 85D	**2**	**A**	**2427**	***Dm***	***Ras85D***	**isotig10293, isotig10294**
rhomboid	**1**	**A**	**1333**	***Dm***	***rho***	**isotig18676**
rolled	**1**	**A**	**728**	***Dm***	***rl***	**isotig25011**
*squid**	1	A	1076	*Dm*	*sqd*	isotig19932
TERMINAL GENES						
arrest	**1**	**A**	**590**	***Dm***	***aret***	**isotig16204**
corkscrew	**2**	**S**	**318-466**	***Dm***	***csw***	**GIB53OK01DRDCY, GIAFTRMO2JG2C8**
hiiragi	2	A	484-637	*Dm*	*hrg*	isotig27382, isotig32450
mago nashi	**2**	**A**	**1230-1235**	***Dm***	***mago***	**isotig10774, isotig10775**
Moesin	1	A	715	*Dm*	*Moe*	isotig25514
nanos	**1**	**A**	**1048**	***Dm***	***nos***	**contig17249**
pumilio	**6**	**S**	**299-406**	***Dm***	***pum***	**GIAFTRM01DD2ST, GIB53OK02FM8FO, GIB53OK01C502X, GIAFTRM01EM7Q9, GIAFTRM02GXICC, GIAFTRM02GHD5G**
Rabenosyn	1	S	412	*Dm*	*Rbsn*	GIAFTRM01CFAVF
Ras1	**2**	**A**	**2427**	***Dm***	***Ras85D***	**isotig10293, isotig10294**
rolled	**1**	**A**	**728**	***Dm***	***rl***	**isotig25011**
torso-like	**1**	**A**	**780**	***Dm***	***tsl***	**isotig14953**
vasa	**1**	**A**	**647**	***Dm***	***vas***	**isotig27190**

LIGANDS, RECEPTORS & EFFECTORS						
18 wheeler	1	A	578	*Dm*	*18w*	isotig28758
argos	1	A	816	*Dm*	*aos*	isotig22917
decapentaplegic	**1**	**A**	**975**	***Dm***	***dpp***	**isotig20685**
hopscotch	**1**	**S**	**314**	***Dm*[2]**	***hop***	**GIAFTRM01CXLBC**
Mothers against dpp	**1**	**S**	**288**	***Dm***	***Mad***	**GIB53OK01B2DPO**
STAT	**1**	**S**	**470**	***Dm***	***Stat92E***	**GAP9EXG05F34AM**
thick veins	**2**	**A, S**	**359-1407**	***Dr***	***bmpr1ba***	**isotig18455, GIAFTRM01C5G7U**
veinlet	**1**	**A**	**1333**	***Dm***	***rho***	**isotig18676**
GENES AFFECTING CYTOSKELETON						
alpha actinin	1	S	455	*Dm*	*Actn*	GIAFTRM01D7QUH
*abnormal spindle**	1	S	473	*Dm*	*asp*	GAP9EXG05F227G
*Bicaudal D**	3	S	341-446	*Dm*	*BicD*	GIAFTRM01A27KC, GIB53OK02F7E1Q, GIB53OK02HFKM7
*Btk family kinase at 29A**	5	S	402-346	*Dm*	*Btk29A*	GIB53OK02FHDVN, GIAFTRM01CKL2U, GIB53OK01EDQ85, GIAFTRM02HZF7J, GIAFTRM01A2C1B
Cdc42	6	A	4142-4277	*Dm*	*cdc42*	isotig02782, isotig02783, isotig02784, isotig02785, isotig02786, isotig02787
Ced-12	1		2258	*Dm*	*ced-12*	isotig17014
*chickadee**	3	A	1018-5120	*Dm*	*chic*	isotig11514, isotig11375, isotig11513
*diaphanous**	1	S	284	*Dm*	*dia*	GIAFTRM01C7J9C
frizzled	**2**	**A, S**	**713**<br>**372**	***Dm***<br>***Dm***	***fz***<br>***fz2***	**isotig25634**<br>**GIB53OK01EXZZC**
genghis khan		S	348	*Dm*[1]	*gek*	GIB53OK02GZ3BD
Kette	3	A, S	338-2119	*Dm*	*Hem*	isotig17258, GIB53OK01AYC1O, GIB53OK01CDNLQ
Lissencephaly-1	3	A	2647-3029	*Dm*	*Lis-1*	isotig07174, isotig07175, isotig07176
Protein kinase A*	**1**	**A**	**1102**	***Dm***	***Pka-C1***	**isotig19762**
*Rac1**	5	A	2613-4872	*Dm*	*Rac1*	isotig02074, isotig02075, isotig02076, isotig02077, isotig02078
rho-type guanine exchange factor	1	S	483	*Dm*	*rtGEF*	GIAFTRM01DOYUC
SCAR	1	A	684	*Dm*	*SCAR*	isotig26362
short stop	5	A, S	370-807	*Dm*	*shot*	GIB53OK02HR0GV, isotig22979, GAP9EXG05FWKKF, GIAFTRM02I8NGU, GIB53OK01EMGN7
spaghetti squash	1	A	735	*Dm*	*sqh*	contig19945
*squid**	1	A	1076	*Dm*	*sqd*	isotig19932
Src oncogene at 64B	1	A	652	*Dm*	*Src64B*	isotig27121

Src oncogene at 42A	3	A	1234-2784	*Dm*	*Src42A*	isotig02154, isotig02151, isotig17454
Suppressor of profilin 2	1	A	1496	*Dm*	*Sop2*	isotig18194
*Tec 29**	5	S	402-346	*Dm*	*Btk29A*	GIB53OK02FHDVN, GIAFTRM01CKL2U, GIB53OK01EDQ85, GIAFTRM02HZF7J, GIAFTRM01A2C1B
*twinstar**	2	A	387-434	*Dm*	*tsr*	contig26952, isotig34793
*zipper**	4	S	423-524	*Dm*	*zip*	GIAFTRM02JY0HB, GIAFTRM01AXRAK, GIAFTRM01ELKZE, GIAFTRM01ENSHU
GENES INFLUENCING CELL CYCLE						
archipelago	1	S	424	*Dm*	*ago*	GIAFTRM01B7E7I
*Btk family kinase at 29A**	5	S	402-346	*Dm*	*Btk29A*	GIB53OK02FHDVN, GIAFTRM01CKL2U, GIB53OK01EDQ85, GIAFTRM02HZF7J, GIAFTRM01A2C1B
Cyclin-dependent kinase 7	1	A	982	*Dm*	*cdk7*	contig27633
Cyclin-dependent kinase subunit 30A	1	S	395	*Dm*	*cks30A*	GIAFTRM02J6WEC
double parked	1	A	641	*Dm*	*dup*	isotig27307
E2F	1	A	1849	*Dm*	*E2F*	contig20027
loki	2	S	301-376	*Dm*	*lok*	GIB53OK01E0SZD, GIAFTRM01AP4KC
*mutagen-sensitive 209**	5	A, S	374-1933	*Dm*	*mus209*	isotig01532, isotig01533, contig44159, GIAFTRM01CN588, GIAFTRM01DK93W
*Myb oncogene-like**	1	A	797	*Dm*	*Myb*	isotig23283
*pitchoune**	3	A	1322-1721	*Dm*	*pit*	isotig18764, isotig10519, isotig10518
Rad51-like	1	A	1335	*Dm*	*Rad51*	isotig18708
twins	1	A	850	*Dm*	*tws*	isotig22216
OTHER GENES INVOLVED IN OOGENESIS						
absent MD neurons & olfactory sensilla	1	S	472	*Dm*	*amos*	GIB53OK02HNBSB
abstrakt	1	S	414	*Dm*[2]	*abs*	GIB53OK02J6WMP
*aubergine**	2	A, S	458-489	*Dm*	*aub*	isotig33798, GIB53OK01DBEHU
Autophagy-specific gene 1	1	S	402	*Dm*	*Atg1*	GIB53OK02H0FDT
basket	**1**	**A**	**5027**	***Dm*[5]**	***bsk***	**isotig16745**
brainiac	8	A	2045-2208	*Dm*	*brn*	isotig00699, isotig00700, isotig00701, isotig00702, isotig00703, isotig00704, isotig00705, isotig00706
bric a brac 1	1	A	478	*Dm*[6]	*bab1*	isotig32822
Buffy	1	A	1077	*Dm*	*Buffy*	isotig19899
*capsuleen**	2	S	412-463	*Dm*	*csu1*	GIAFTRM02G9BFL, GIB53OK02IE8IK
Calmodulin-binding protein related to a	3	S	304-453	*Dm*	*crag*	GIAFTRM02GJRJS, GIB53OK02GW9JP,

Rab3 GDP/GTP exchange protein						GIB53OK01CQUNA
cornichon	**4**	**A**	**1304-1876**	***Dm***	***cni***	**isotig05798, isotig05799, isotig05800, isotig05801**
Death caspase-1	1	S	499	*Dm*	*Dcp1*	GIB53OK01EBR79
discs large	2	S	400-479	*Dm*	*dlg1*	GIB53OK01EFPOT, GIB53OK01CQL58
Ecdysone-induced protein 63E	2	S	450-524	*Dm*[1]	*Elp63E*	GIAFTRM01EDEJS, GIAFTRM01EAC5V
eggless	4	S	314-406	*Dm*	*egg*	GIAFTRM01D7QDL, GIB53OK02IV6JT, GIB53OK01D738W, GIB53OK02JRHEF
extra macrochaetae	1	A	2306	*Dm*[3]	*emc*	isotig17133
14-3-3 epsilon	1	S	348	*Dm*	*14-3-3e*	GIB53OK02JB1FY
fringe	**4**	**A**	**3395-3399**	***Dm***	***fng***	**isotig05593, isotig05594, isotig05595, isotig05596**
G protein-coupled receptor kinase	3	S	434-468	*Dm*[1]	*Gprk2*	GIAFTRM02FRLLL, GIAFTRM02F24EF, GIAFTRM01A46K8
+G protein alpha 47A	4	A	2035-2037	*Dm*	*G-alpha47a*	isotig06627, isotig06628, isotig06629, isotig06630
half pint	3	S	335-512	*Dm*	*puf68*	GIAFTRM02IXFZ3, GIAFTRM02HZRK5, GIB53OK01EHMPZ
*Heat shock factor**	1	A	2157	*Dm*	*Hsf*	isotig06696
Heat shock protein 70	1	A	2435	*Dm*	*Hsp70Ab*	isotig17067
*hephaestus**	1	A	481	*Dm*	*heph*	isotig32718
hunchback	1	A	1298	*Dm*	*hb*	isotig18835
*Ice**	1	S	453	*Dm*[4]	*Ice*	GIAFTRM01EEGBL
Jun related antigen	**1**	**A**	**1744**	***Dr***	***jun***	**isotig17712**
leonardo	2	A	176-469	*Dm*	*14-3-3z*	contig19016, contig19014
Liprin-a	4	S	382-507	*Dm*	*Liprin-a*	GIB53OK01BN50J, GIAFTRM01A7ILZ, GIB53OK01DKAGG, GIB53OK01AKTBO
maternal expression at 31B	1	A	734	*Dm*	*me31B*	contig28380
microtubule star	**2**	**A, S**	**485-855**	***Dm***	***mts***	**GIAFTRM01DK761, isotig22169**
mini spindle	2	S	264-294	*Dm*	*msps*	GIAFTRM01BGE9N, GIAFTRM02JNY0C
mirror	1	A	824	*Dm*	*mirr*	isotig22803
misshapen	1	S	479	*Dm*	*msn*	GIB53OK01E2GV6
moira	1	A	477	*Dm*	*mor*	isotig32460
*mutagen-sensitive 209**	5	A, S	374-1933	*Dm*	*mus209*	isotig01532, isotig01533, contig44159, GIAFTRM01CN588, GIAFTRM01DK93W
nicastrin	**1**	**A**	**2422**	***Dm***	***nct***	**contig00347**
Niemann Pick Type C-2a	2	A	815-1293	*Dm*	*Npc2a*	contig35173, isotig11294
*Nucleolar protein at 60B**	1	A	1626	*Dm*	*Nop60B*	isotig17924

O-fucosyltransferase 1/neurotic	1	A	2713	*Dm*	*O-fut1*	isotig06103
Ornithine decarboxylase antizyme	5	A	2605-2655	*Dm*	*Oda*	isotig00265, isotig00266, isotig00267, isotig00268, isotig00269
Pcaf	3	A, S	323-1184	*Dm*	*Pcaf*	isotig30852, GIAFTRM02F20GN, isotig19282
*pitchoune**	3	A	1322-1721	*Dm*	*pit*	isotig18764, isotig10519, isotig10518
polyhomeotic proximal	1	S	510	*Dm*	*ph-p*	GIAFTRM01BODQE
Presenilin	**1**	**S**	**276**	***Dm***	***Psn***	**GIAFTRM02H10N7**
puckered	1	A	1025	*Dm*	*puc*	isotig20292
Rab-protein 5	2	A	4229-4232	*Dm*	*Rab5*	isotig07186, isotig07187
*Rab-protein 11**	1	A	945	*Dm*	*Rab11*	isotig20992
*sans fille**	4	A	289-941	*Dm*	*snf*	isotig13572, isotig13573, contig63129, contig63131
scribbled	2	A, S	378-469	*Dm*	*scrib*	isotig33249, GIAFTRM02GAT19
SNF1A/AMP-activated protein	1	A	1679	*Dm*	*SNF1A*	isotig17824
Snf5 related 1	1	A	786	*Dm*	*snr1*	isotig23635
spinster	2	A	516-987	*Dm*	*spin*	isotig20576, isotig30779
strawberry notch	6	S	329-411	*Dm*	*sno*	GIB53OK01ESFGF, GIB53OK02IHK84, GIAFTRM02JXN3Y, GIB53OK01DUES8, GIAFTRM02JY5R1, GIB53OK01E1H5S
Suppressor of variegation 3-3	1	A	767	*Dm*	*Su(var) 3-3*	isotig24052
Syntaxin 1A	2	A	905-1424	*Dm*	*Syx1A*	isotig18436, contig12794
*TATA box binding protein-related factor 2**	1	A	1030	*Dm*	*Tfr2*	isotig20178
TBP-associated factor 1	4	A, S	350-1227	*Dm*	*Taf250*	isotig19131, isotig26557, GIAFTRM01DQZ6B, isotig23792
twin		A	533	*Dm*	*twin*	isotig30050
yan	**1**	**A**	**691**	***Dm***	***aop***	**isotig26139**
widerborst	**2**	**A**	**874-1143**	***Dm***	***wdb***	**isotig19487, isotig21842**
